# The core fungal microbiome of banana (*Musa* spp.)

**DOI:** 10.3389/fmicb.2023.1127779

**Published:** 2023-03-30

**Authors:** Henry W. G. Birt, Anthony B. Pattison, Adam Skarshewski, Jeff Daniells, Anil Raghavendra, Paul G. Dennis

**Affiliations:** ^1^School of Earth and Environmental Sciences, The University of Queensland, Brisbane, QLD, Australia; ^2^Department of Agriculture and Fisheries, Centre for Wet Tropics Agriculture, South Johnstone, QLD, Australia

**Keywords:** fusarium, fungal diversity, plant-microbe interactions, mycobiome, network, holobiont

## Abstract

Here, we report a metabarcoding (ITS2) study to define the common core fungal microbiome (mycobiome) of healthy *Musa* spp. (bananas and plantains). To identify a list of 21 core fungal taxa, we first characterised the effects of edaphic conditions and host genotype – two factors that are likely to differ between farms – on the diversity of fungal communities in bulk soil and seven plant compartments. This experiment facilitated shortlisting of core ‘candidates’, which were then elevated to full core status if also found to frequent a wide-range of field-grown *Musa* spp. and exhibit hub-like characteristics in network analyses. Subsequently, we conducted a meta-analysis of eleven publicly available datasets of *Musa* spp. associated fungi demonstrating that the core fungi identified in our study have close relatives in other countries. The diversity and composition of mycobiomes differed between plant compartments and soils, but not genotypes. The core mycobiome included *Fusarium oxysporum* and its relatives, which dominated all plant compartments, as well as members of the *Sordariomycetes*, *Dothideomycetes*, and *Mortierellomycota*. Our study provides a robust list of common core fungal taxa for *Musa* spp. Further studies may consider how changes in the frequencies and activities of these taxa influence host fitness and whether they can be managed to improve banana production.

## Introduction

*Musa* spp. (bananas and plantains) are one of the world’s most important fruit crops but are constrained by a range of abiotic and biotic stresses, including diseases for which management options are either unavailable, or becoming less effective (Gutierrez-Monsalve et al., 2015; Bubici et al., 2019; Fu et al., 2019). Fungal diseases of *Musa* spp., such as black leaf streak, Eumusae leaf spot, freckle, and Fusarium wilt, are particularly notorious; however, many fungi benefit host fitness (Yano-Melo et al., 2003; Rodríguez-Romero et al., 2005; Drenth and Kema, 2021). If managed appropriately, banana fungal microbiomes (mycobiomes) could help growers to maintain healthy production systems. Nonetheless, plant-associated fungi are extremely diverse and may vary between locations. Hence, it is important to identify fungal taxa that are persistently associated with *Musa* spp. across a wide range of environmental conditions, *viz.* the ‘common core’ (Risely, 2020). This approach emphasises a relatively small subset of taxa on which to focus research efforts and helps avoid the development of microbiome management approaches that are context dependent (Toju et al., 2018). A common core mycobiome of *Musa* spp. is yet to be defined.

Different plant compartments offer unique niches for fungi and should be considered when defining a core mycobiome (Cregger et al., 2018). Previous studies of fungi associated with *Musa* spp. have focused on below-ground interactions, leaving the mycobiomes of above-ground plant compartments relatively underexplored (Wang et al., 2015; Rames et al., 2018; Shen et al., 2018). The diversity and composition of bacterial communities associated with *Musa* spp. have been shown to differ greatly between plant compartments (Birt et al., 2022). However, for fungi this association may be weaker. In cereals, legumes, *Brassicaceae*, and *Agave* spp., for example, the location in which plants are grown, rather than plant compartment has been observed to have a larger impact on the diversity and composition of fungal communities (Coleman-Derr et al., 2016; Tkacz et al., 2020).

Edaphic factors also need to be considered when defining a core mycobiome of *Musa* spp. as soil is the primary source of fungi that colonise plants (Busby et al., 2017). The diversity and composition of soil fungal communities differs depending on both abiotic factors such as nutrient availability (Vasco-palacios and Bahram, 2020) soil structure (Xia et al., 2020), and biotic factors such as plant-mediated soil feedback (Raaijmakers and Mazzola, 2016). Additionally, edaphic factors may also influence the types of relationships fungi play within the mycobiome ranging from pathogens to mutualists (Guerrero-Ariza and Posada, 2017). Hence, fungi that persistently associate with *Musa* spp. across diverse edaphic conditions are likely to be relevant to a range of production sites.

While the effects are often smaller than those associated with soil properties, the impacts of host genotype should also be considered when defining a core *Musa* spp. mycobiome (Hannula et al., 2010; Laurent et al., 2010). Changes in hormones, tissue phenotype, and life cycle timing between various genotypes can influence host-associated fungi (Vorholt et al., 2017). Finding a common core between *Musa* genotypes is important because it is estimated that there are more than 500 cultivars in use globally (Stover and Simmonds, 1987).

Here, we characterised the common core mycobiome of *Musa* spp. Our study began with a pot experiment to determine the impacts of plant compartment, edaphic conditions, and host genotype on fungal diversity using ITS2 rRNA gene amplicon sequencing. This experiment comprised more than 480 samples from eight plant compartments, three genotypes, and five distinct soils, and was used to define a list of candidate-core fungal taxa. We then characterised the fungal communities associated with more than 400 samples from field-grown *Musa* spp. comprising 52 genotypes. These results were used to refine our list of candidates and identify a final set of common core fungal taxa, which were compared with other members of the microbiome using network analysis, including bacteria from a previously published study (Birt et al., 2022). Finally, by comparing the sequences of our core fungi with those reported in 11 previous studies of banana-associated fungi, we provide evidence that they are commonly associated with *Musa* spp. in other parts of the world.

## Methods

### Experimental design

*Pot experiment:* To investigate the potential impacts of plant compartment, soil, and genotype on *Musa* spp. mycobiomes we conducted a pot experiment in a glasshouse as described previously in Birt et al. (2022). Briefly, the banana variety ‘Williams’ *Musa* (AAA Group, Cavendish Subgroup), Australia’s most common commercial cultivar (Australian Banana Growers Council, 2021) was grown in five distinct soils collected (0–30 cm depth) from North Queensland, Australia’s primary banana producing region (Supplementary Table S1). In one of these soils only, we also grew the banana variety ‘Lady finger’ *Musa* (AAB Group, Pome Subgroup) and the banana variety ‘Goldfinger’ *Musa* (AAAB Group, Prata Anã x SH-3142) to investigate the effect of genotype on *Musa* spp. mycobiomes. These genotypes represented Australia’s second most common cultivar (Australian Banana Growers Council, 2021) and a Fusarium wilt resistant cultivar (De Ascensao and Dubery, 2000), respectively. Eight plant compartments were sampled using an established procedure (Birt and Dennis, 2021): bulk soil (BS), the ectorhizosphere at the apex and base of the roots (AER, BER), the endorhizosphere at the apex and base of the roots (AEnR, BEnR), the rhizome/corm (C), the pseudostem (PS), and leaves (L) (Supplementary Figure S1).

Having assessed the key drivers of fungal communities associated with *Musa* spp. and used these to define a core mycobiome, we also performed a field survey to confirm our findings under field conditions and in a wider range of genotypes. Bulk soil (BS), ectorhizosphere (ER), endorhizosphere (EnR), pseudostem (PS), and leaf (L) samples were collected from 55 plants, representing 52 genotypes in a single field from the Australian *Musa* germplasm collection, South Johnstone, Queensland (Supplementary Table S2). For each plant, we collected a set of samples (i.e., BS, ER, EnR, PS, and L) associated with a young (large sucker), medium (emerged adult stem in vegetative growth), and older (signs of active flowering) pseudostem to provide three replicates for each compartment and encompass variation that may be attributable to the age of various tissues. Strict biosecurity is in place at the site and all plants appeared healthy with no signs of pest or pathogen pressure.

### DNA extraction and fungal community profiling

*DNA extraction:* All samples were lyophilised, homogenised by grinding, and then DNA was extracted using the high throughput Power Soil DNA Isolation kit (Qiagen) according to the manufacturer’s instructions except for an extra 400 μl of Powerbead solution to moisten freeze dried samples enough for efficient extraction.

*PCR*: To avoid host co-amplification, we used the primers ITS-F_KYO1 (5′- CTH GGT CAT TTA GAG GAA STA A-‘3) (Toju et al., 2012) and ITS4 (5’- TCC TCC GCT TAT TGA TAT GC-‘3) (White et al., 1990) in polymerase chain reactions (PCRs) to amplify the full ITS region (PCR 1). These regions are less conserved among fungi and plants and therefore avoid plant-associated reads. We then purified the products using magnetic beads (Rohland and Reich, 2011) and used these as template in a second PCR targeting the ITS2 region using the more universal primers gITS7 (5’- GTG AAT CAT CGA ATC TTT G-‘3) (Ihrmark et al., 2012) and ITS4 to obtain an amplicon of appropriate length for DNA sequencing (PCR 2). In the second PCR, both primers were modified on the 5’ end to contain the Illumina overhang adapter for compatibility with the P5 and i7 Nextera XT indices. We tested the impact of this nested PCR (full ITS, then ITS2) on fungal community composition relative to a single step PCR (ITS2 only) and found no significant difference between protocols (see Supplementary experiment).

Thermocycling conditions were as follows: 98°C for 45 s; then 15 cycles for PCR 1 or 20 cycles for PCR 2 of 98°C for 5 s, 56°C for 5 s, 72°C for 6 s; followed by 72°C for 1 min. Amplifications were performed using a SimpliAmp^®^ 96-well Thermocycler (Applied Biosystems). All PCRs were performed on 2 μl template in 5X Phire Green Reaction Buffer (Thermo Fisher), 100 μM of each dNTP (Invitrogen), 0.4 μl Phire Green Hot Start II DNA Polymerase (Thermo Fisher), 10 mM of each primer, made up to a total volume of 20 μl with molecular biology grade water. Amplicons were purified and then dual indexed using the Nextera XT Index Kit (Illumina) according to the manufacturer’s instructions. Indexed amplicons were then purified, quantified using a PicoGreen dsDNA Quantification Kit (Invitrogen), and then pooled in equimolar concentrations prior to being sequenced on an Illumina MiSeq using 30% PhiX Control v3 (Illumina) and a MiSeq Reagent Kit v3 (600 cycles, Illumina) according to the manufacturer’s instructions.

### Processing of sequence data

By combining all data from the pot experiment and field survey into a single bioinformatic analysis, we were able to compare the two datasets to validate whether core taxa identified in pots were present in the field. Data was processed by using a modified UPARSE workflow (Edgar, 2013). Firstly, samples were demultiplexed using cutadapt from QIIME2 (v2017.9.0, Bolyen et al., 2019). Fungal ITS2 regions were then extracted using ITSx (v1.0.11, Bengtsson-Palme et al., 2013) and chimeric sequences were removed using uchime2_ref of USEARCH (v10.0.240, Edgar, 2010) against the UNITE 8.2 database (Nilsson et al., 2019). The resulting reads were then mapped against representative sequences using fastx_uniques and cluster_otus (sequence similarity = 0.97) from USEARCH to produce an OTU table. BLASTN (Zhang et al., 2000) from QIIME2 was used to assign taxonomy using the UNITE 8.2 database (Nilsson et al., 2019). Samples were rarefied to 1,000 reads per sample. QIIME2 was used to calculate all alpha diversity metrics. While our nested PCR approach greatly reduced host contamination, *Musa* ITS2 rRNA gene reads were still present in some samples; hence, some samples had fewer than 1,000 fungal reads and were discarded. While most treatment combinations retained all 10 replicates, some ended up with fewer, albeit not less than four (Supplementary Tables S3–S5).

### Statistical analyses

*Effects of compartment and soil/genotype:* Differences in alpha diversity (observed OTUs, Chao1, and Shannon) were investigated using ANOVA with Tukey’s HSD for *post hoc* analyses. Differences in fungal community composition between treatments (i.e., beta diversity) were investigated using PERMANOVA as implemented in the R package *vegan* (Oksanen et al., 2017). Fungal OTU relative abundances were Hellinger transformed prior to analysis. Differences in the composition of fungal communities associated with treatments shown to be significant were visualised using redundancy analysis (RDA) in *vegan*. All statistical analyses were performed using R version 3.6.0.

SourceTracker, a Bayesian statistical tool (Knights et al., 2011), was used to examine the extent to which the microbial community of a plant compartment was sourced from other compartments. Each compartment was defined as a microbial source and was compared to all others when using SourceTracker. A grand mean and standard deviation were calculated after SourceTracker had been run on the data from plants grown in each representative soil.

*Definitions of core taxa:* Abundance and occupancy relationships were chosen to define core OTUs as these are grounded in macroecological theory as a tool to establish the range of a species (Hanski, 1982; Gaston et al., 2000). Prevalent and abundant OTUs were defined as those present in ≥50% of samples within a plant compartment with an average relative abundance of ≥0.5% where found. These values were chosen as the minimum for defining a core OTU as they represent a level that minimises stochastic association (through prevalence thresholds) and to circumvent bias in the estimation of variability for taxa with low abundance as they would be near our detection limit. Studies examining core microbes in a range of other environments have taken similar approaches (Barnett et al., 2015; Brodie et al., 2016; Billiet et al., 2017; Adam et al., 2018). Candidate-core OTUs were identified based on those that were found within plants grown in all five soils.

Next, a final list of core taxa was produced by removing those only found in the pot dataset as these taxa were likely to occur due to the differences between the pot and field-grown plants. This was achieved by removing taxa that were not in the list of taxa found in the field dataset that were ‘key constituents’, defined as found in ≥50% of samples at an abundance of ≥0.5%. Through this validation process, we were able to confirm that a more stringent definition of core OTUs in the pot experiment (beyond ≥50% of samples within a plant compartment or beyond an average relative abundance of ≥0.5% where found) would miss some abundant and prevalent OTUs present in both datasets (Supplementary Figure S2).

### Network analysis

Weighted co-occurrence networks were created from both the fungal and bacterial OTU tables from this and our previous study on the bacterial microbiome of *Musa* spp. (Birt et al., 2022), using samples from the independent field survey. Only OTUs found to have ≥1% relative abundance in three samples were included in the analysis to reduce computational load. Networks were calculated in R using the multi.spiec.easi function in the *SpiecEasi* package that accounts for the use of two independent but compositional datasets that are used in a single network (Kurtz et al., 2015; Tipton et al., 2018). The resulting network was then projected using Gephi (Bastian et al., 2009). Degree, betweenness centrality, Markov centrality, and closeness centrality were calculated using the *igraph* and *centiserve* R packages (Csardi and Nepusz, 2006; Jalili, 2017). A *Fusarium* sub-network and the corresponding centrality metrics were created and calculated using Gephi. All centrality metrics were assessed between candidate-core and non-core taxa using Wilcoxon rank-sum tests implemented in base R.

### Meta-study of publicly available data

To determine the applicability of our core mycobiome to a worldwide context, we downloaded data from 11 previous phylogenetic marker gene studies on fungi associated with *Musa* spp. (Supplementary Table S6) using the SRA explorer (Ewels, 2020). For ITS rRNA gene high-throughput sequencing studies, the ITS1 and ITS2 regions were extracted using ITSx (Bengtsson-Palme et al., 2013). For 18S data, adapters were removed using cutadapt (Martin, 2011). Sequences were then filtered to remove low-quality reads, and an OTU table was produced using USEARCH (Edgar, 2013). The representative sequences were extracted from the top 10% of OTUs sorted by the maximum seen in any sample. BLASTN (Zhang et al., 2000) from QIIME2 was again used to assign taxonomy using against the UNITE 8.2 database for ITS rRNA gene sequences and SILVA 128 for 18S rRNA gene sequences (Quast et al., 2012; Nilsson et al., 2019). Next, the top hit based on the highest e-value was extracted for each OTU from the blast results. The taxonomy assigned for core and candidate-core OTUs was then searched for in these results using a custom R script.

## Results

### The effects of compartment, soil, and host genotype within the pot experiments

**Figure 1:**
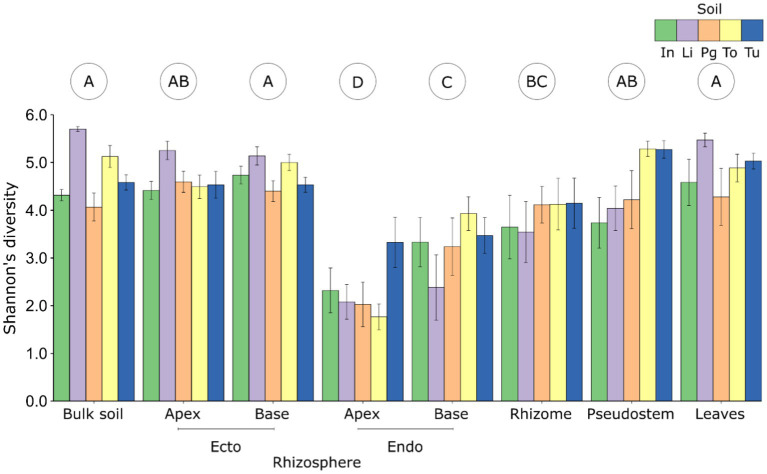
The alpha diversity (numbers of observed OTUs) of fungal communities associated with different plant compartments of *Musa* (AAA Group, Cavendish Subgroup) ‘Williams’ grown in pots with five distinct soils. Error bars represent standard errors of the means. Letters in circles indicate compartments that differ according to Tukey *post hoc* tests. endo, endorhizosphere; ecto, ectorhizosphere; In, Innisfail; Li, Liverpool; Pg, Pin Gin; To, Tolga; Tu, Tully.

**Figure 2:**
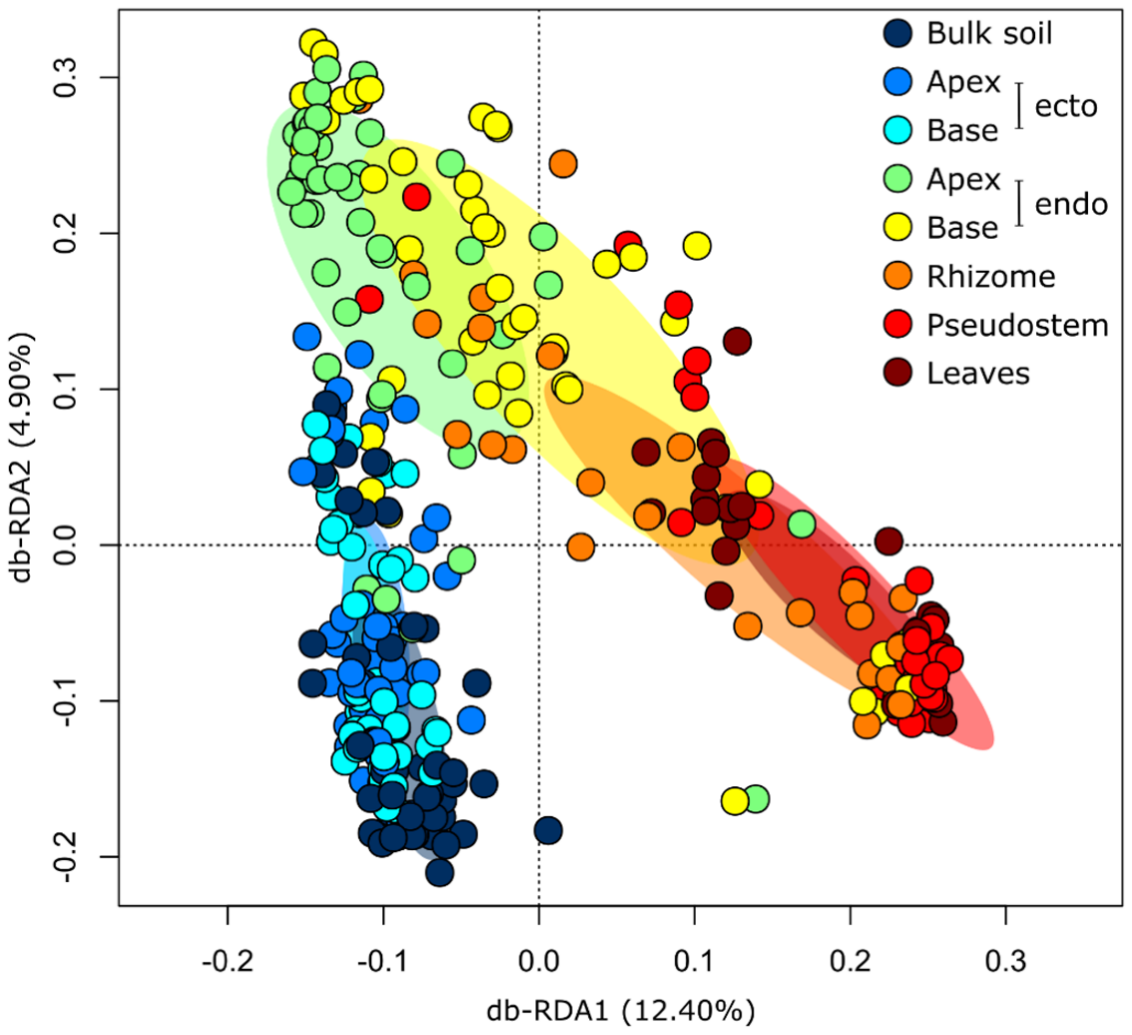
Distance-based Redundancy Analysis (RDA) ordination highlighting differences in the composition of fungal communities (Hellinger transformed OTUs) associated with *Musa* (AAA Group, Cavendish Subgroup) ‘Williams’ in various plant compartments. The ellipses represent standard deviations of the group centroids. endo, endorhizosphere; ecto, ectorhizosphere.

*Compartment:* The diversity and composition of fungal communities differed significantly between plant compartments, and this effect was stronger than those of soil and host genotype (Table 1; Supplementary Table S7). The least diverse fungal communities were associated with the apical endorhizosphere, followed by the basal endorhizosphere, the rhizome (Figure 1). The pseudostem and leaves were as diverse as the ectorhizosphere and bulk soil (Figure 1). In terms of composition, fungal communities were more similar in compartments that were closer to one another, with bulk soil and ectorhizosphere communities being distinct from those associated with endorhizosphere and above-ground plant compartments (Figure 2). This finding was also supported by Bayesian estimates of community provenance, which indicated that 91% of the pseudostem fungal microbiome was sourced from leaves, in contrast to 62% from bulk soil (Supplementary Table S8).

**Table 1:** The impacts of soil, genotype, and plant compartment on the alpha diversity (Shannon’s Diversity Index) and composition (Hellinger transformed OTUs) of fungal communities using ANOVA and PERMANOVA, respectively.

Predictor variable		Shannon’s diversity		Community composition		
	*df*	*F* value	*p* value	*F* value	*R*^2^ (%)	*p* value
Compartment	7	30.3	<0.001***	15.5	21.5	<0.001***
Soil	4	2.8	0.028*	7.4	5.9	<0.001***
Compartment: Soil	28	1.7	0.021*	2.0	11.2	<0.001***
Compartment	7	14.5	<0.001***	12.1	29.3	<0.001***
Genotype	2	0.5	0.596	1.4	1.0	0.074
Compartment: Genotype	14	0.7	0.770	1.1	5.2	0.221

These results derive from our pot experiment which included five distinct soils, three *Musa* spp. genotypes, and eight compartments.

**Figure 3:**
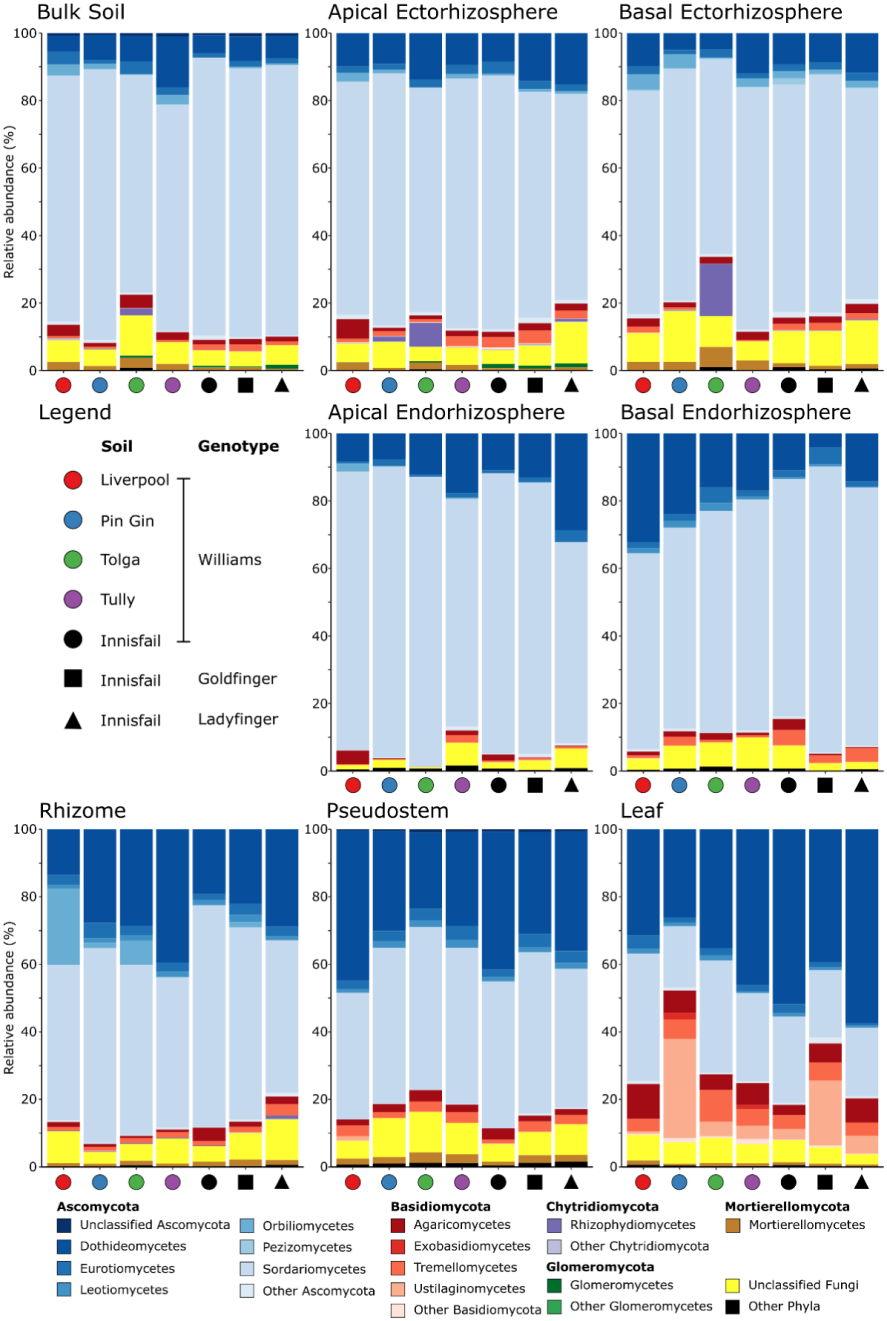
The mean relative frequencies of fungal classes in different plant compartments associated with *Musa* (AAA Group, Cavendish Subgroup) ‘Williams’ grown in pots with five distinct soils, and two other *Musa* spp. genotypes grown in pots containing an Innisfail series soil. Within each phylum, classes represented at <1% mean relative abundance are grouped as other.

Members of the *Ascomycota* formed the majority of reads in all *Musa* spp. compartments (Figure 3). They were most abundant in the apical endorhizosphere (94.3% mean relative abundance, Figure 3) and least abundant in the leaves (70.8% mean relative abundance, Figure 3). While present in other compartments, representatives of the Basidiomycota were most common in leaves, where they comprised 10.5–45.0% mean relative abundance (Figure 3). Members of the *Mortierellomycota* and *Glomeromycota* were also detected but were relatively infrequent (Figure 3). Furthermore, c. 6.6% mean relative abundance of fungi in all communities could not be allocated a taxonomic rank below kingdom (Figure 3). Finally, while negligible in plants grown in other soils, members of the Chytridiomycota represented 11.3% mean relative abundance within the ectorhizosphere of plants grown in Tolga soil (Figure 3).

*Soil and genotype*: The diversity and composition of fungal communities also differed significantly between soils but not genotypes (Tables 1, 2). According to the Shannon Index, fungal diversity differed significantly between soils in the pseudostem, basal ectorhizosphere, and bulk soil (Table 2); however, these differences were not supported by all alpha diversity metrics (Supplementary Table S7). Fungal community composition was observed to significantly differ between soils in all belowground compartments, except the rhizome (Table 2).

**Table 2:** The influence of soil on the alpha diversity (Shannon’s Diversity Index) and composition (Hellinger transformed OTUs) of fungal communities within each compartment, as assessed by ANOVA and PERMANOVA, respectively.

Compartment	Shannon diversity		Community composition		
	*F* value	*p* value	*F* value	*R^2^* (%)	*p* value
Bulk soil	12.7	<0.001***	6.7	38.5	<0.001***
Apical ectorhizosphere	2.2	0.083	4.5	28.4	<0.001***
Apical endorhizosphere	1.9	0.123	1.9	14.9	<0.001***
Basal ectorhizosphere	2.8	0.038*	5.6	33.6	<0.001***
Basal endorhizosphere	1.1	0.367	1.7	13.7	0.004**
Rhizome	0.2	0.912	1.3	14.3	0.169
Pseudostem	2.9	0.037*	1.5	14.3	0.086
Leaf	1.2	0.345	1.3	16.9	0.123

The results are for the *Musa* (AAA Group, Cavendish Subgroup) ‘Williams’ plants grown in five distinct soils in our pot experiment.

### Identifying ‘candidate core’ fungal taxa

Given that compartment and soil, but not genotype, were found to influence the *Musa* spp. mycobiome, we defined a list candidate-core fungal OTUs as follows. Firstly, for each soil, we identified the most abundant and prevalent OTUs on the basis that they were found in ≥50% of replicates one or more compartments, at a mean relative abundance of ≥0.5% where present (Supplementary Figures S3, S4). From the 173 OTUs that met these criteria, we then removed any that were not found in all soils (Supplementary Figures S3–S5), leaving 42 that were given ‘candidate-core’ status (Supplementary Figure S5).

### Refining the ‘core’ mycobiome of *Musa* spp.

Next, we surveyed the fungal microbiomes of 52 field-grown banana plants, to assess whether the ‘candidate-core’ fungal OTUs identified in our pot experiment were also important under more realistic conditions. As observed in pots, field-grown plants were dominated by members of the Ascomycota and Basidiomycota (Supplementary Figure S6).

The ‘importance’ of ‘candidate-core’ fungal OTUs was considered from two key perspectives. Firstly, we inferred their importance to putative microbiome interactions using network analyses. These interactions were inferred using SPIEC-EASI for fungal ITS2 data only, and fungal ITS2 in combination with bacterial 16S data from the same samples (Birt et al., 2022; Supplementary Figures S7, S8). In both cases, ‘candidate-core’ fungal OTUs had significantly more connections (Degree), and larger betweenness, closeness, and Markov centrality scores than non-core OTUs (Table 3).

**Table 3:** Results from Wilcoxon sum rank tests of centrality metrics from fungal OTUs considered core and non-core.

Network	Metric	Candidate-core fungi	Non-core fungi	*W*	*p*
		(Median 1st quartile, 3rd quartile)	(Median 1st quartile, 3rd quartile)		
Fungi	Betweenness	184 (42, 793)	30 (0, 181)	2,760	0.002**
Fungi	Closeness	1.98*10^−4^ (1.95*10^−4^, 2.00*10^−4^)	1.96*10^−4^ (1.87*10^−4^, 1.99*10^−4^)	2,640	0.011*
Fungi	Degree	4 (3, 8)	3 (1, 6)	2,537	0.033*
Fungi	Markov	7.06*10^−4^ (6.0*10^−4^, 7.8*10^−4^)	6.56*10^−4^ (3.9*10^−4^, 7.5*10^−4^)	2,575	0.024*
Fungi and bacteria	Betweenness	398 (193, 1,031)	149 (34, 381)	2,978	<0.001***
Fungi and bacteria	Closeness	2.64*10^−4^ (2.58*10^−4^, 2.70*10^−4^)	2.56*10^−4^ (2.50*10^−4^, 2.63*10^−4^)	2,828	<0.001***
Fungi and bacteria	Degree	8 (6, 11)	6 (3, 8)	2,719	0.004**
Fungi and bacteria	Markov	1.33*10^−3^ (1.0*10^−3^, 1.7*10^−3^)	2.56*10^−4^ (2.5*10^−4^, 2.6*10^−4^)	2,695	0.006**

Letters indicate OTUs included in the network: F are fungi only, F and B are fungi and bacteria. Networks were built from data from an independent field survey of 52 genotypes of *Musa* spp.

**Figure 4:**
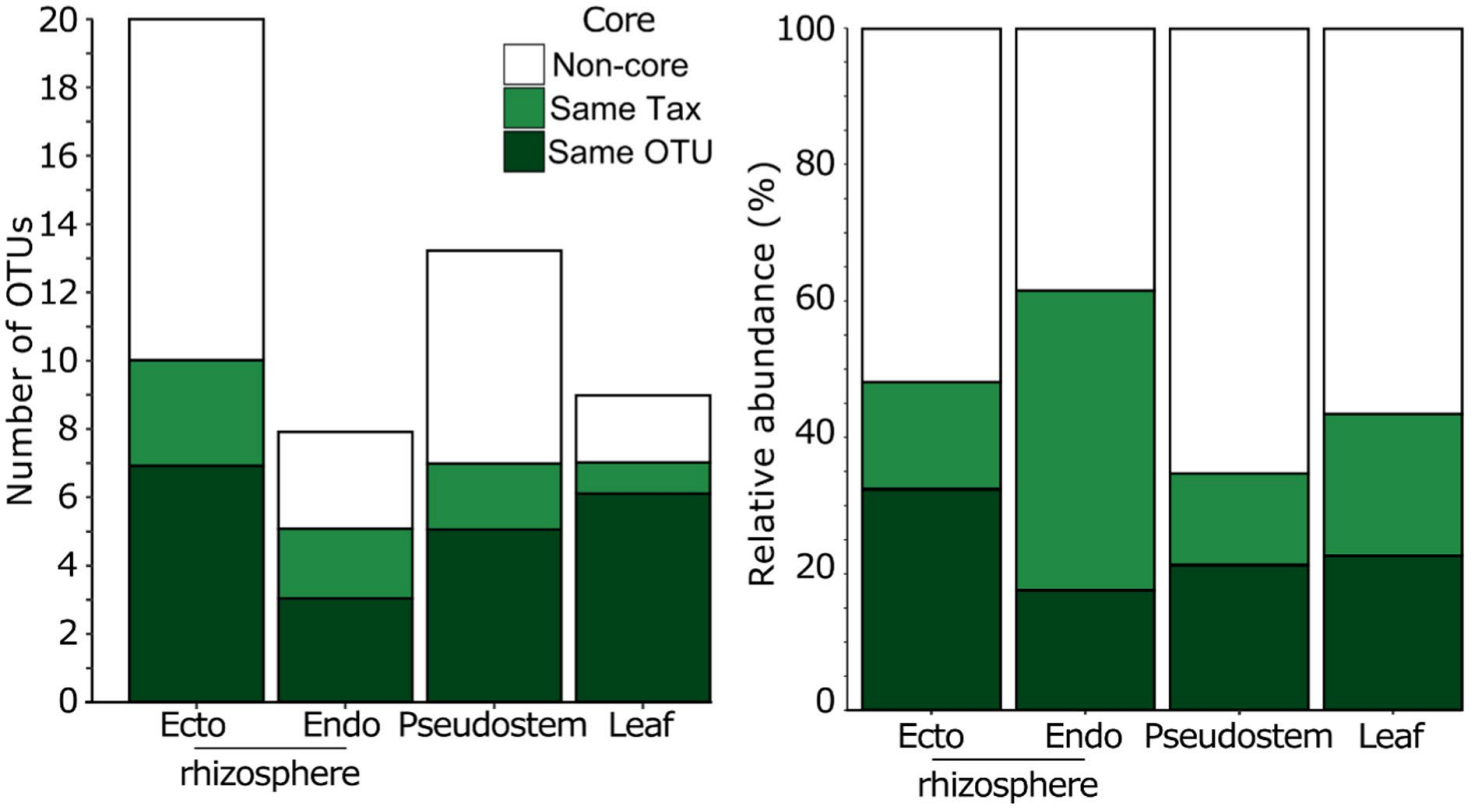
The number of abundant and prevalent OTUs from the field experiment and the relative abundance of fungi that were defined as core or non-core in each plant compartment from 52 field grown *Musa* spp. genotypes.

Secondly, we inferred the importance of candidate core OTUs based on whether they were represented among the most abundant and prevalent fungal OTUs in the field mycobiomes (i.e., those present in ≥50% of field-grown plants at ≥0.5% mean relative abundance). Of the 36 OTUs that met these criteria, 14 were classified as ‘candidate core’ OTUs (i.e., same OTU), and seven were considered close relatives (i.e., identical taxonomy but different OTUs). Together, these 21 OTUs were elevated to full ‘core’ status and represented the majority of the most abundant and prevalent OTUs in every compartment (Figure 4). In addition, while representing only 0.35% of all OTUs in the field, the 21 ‘core’ OTUs represented c. 50–60% and c. 35–45% of sequences in roots and above-ground compartments, respectively (Figure 4). Lastly, 95% (20/21) of ‘core’ OTUs were represented in all 52 *Musa* spp. genotypes examined, either as the same OTU or a close relative. The only exception was *Phaeosphaeria oryzae,* which was present in 87% (45/52) *Musa* spp. genotypes.

### The core mycobiome of *Musa* spp.

**Figure 5:**
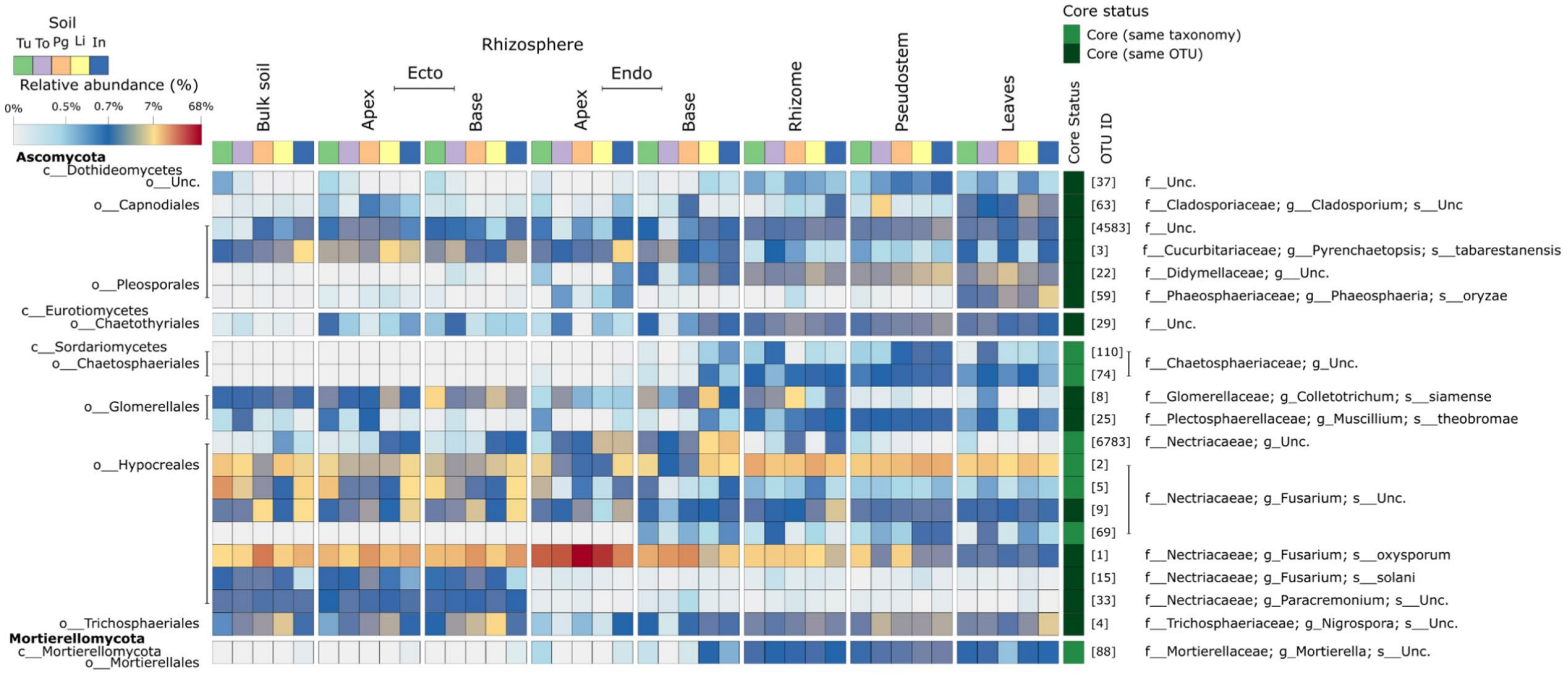
A heatmap showing the mean abundance of the core fungal microbiome taxa identified in this study in various *Musa* (AAA Group, Cavendish Subgroup) ‘Williams’ plant compartments and plants grown in distinct soils.

The 21 core fungal OTUs constitute nine distinct genera, including eight within the Ascomycota, and one within the *Mortierellomycota* (Figure 5; Supplementary Figure S9). Representative ITS2 gene sequences of the core candidate-core are provided in the supplementary information (Supplementary Table S9).

*Fusarium* spp. and their close relatives represent a significant proportion of the final core. Eight (38%) of the final core are members of the *Nectriacaeae* – the family containing *Fusarium* spp. OTU 1 (*Fusarium oxysporum*) was found at a high relative abundance throughout the plant and represented >50% of reads in the apical endorhizosphere (Figure 5). Core taxa that were not identified as *Fusarium* spp. were not found throughout the entire plant (Figure 5; Supplementary Figure S8). Different core taxa occupied the root-associated compartments compared to other compartments. For example, *P. oryzae* and *Cladosporium* sp. were found to be dominant only in the phyllosphere (Figure 5; Supplementary Figure S7). *Pyrenchaetopsis tabarestanensis* was dominant only in the root-associated compartments and bulk soil (Figure 5; Supplementary Figure S8).

**Figure 6:**
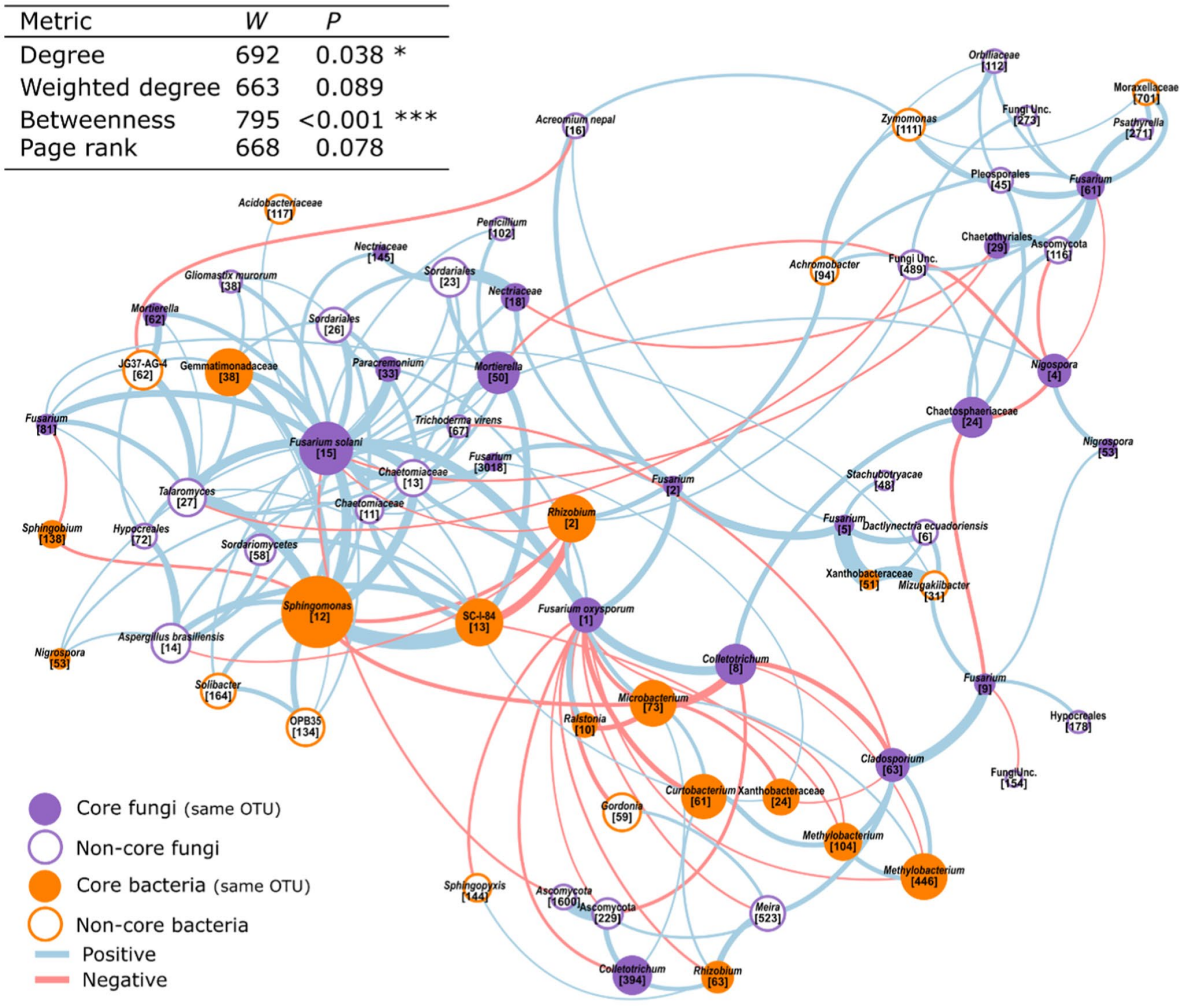
A subset of a dual amplicon co-occurrence network showing the interactions between Fusarium OTUs and their first order neighbours. Those that are core bacteria or fungi are highlighted. This network was created from the microbiome of 52 adult *Musa* spp. in a field setting. Node size is scaled by weighted degree.

Next, we used our microbiome networks to infer the putative interactions of core- taxa with *Fusarium* spp., which are known to influence the health of *Musa* spp. (Alabouvette, 1999; Nel et al., 2006a; Hill et al., 2021). Within this subnetwork, core taxa formed 46% of the nodes. Furthermore, core taxa were significantly more central according to the number of connections made (degree) and their tendency to connect distinct parts of the network (betweenness, Figure 6).

### An assessment of the core taxa in publicly available datasets

Finally, we sought to determine whether our core fungal taxa had been detected in association with banana plants in other parts of the world. To do this, we used publicly available data from 11 other studies that considered the *Musa* spp. mycobiome (Supplementary Table S6). These studies expanded our search to China, Uganda, and other parts of Australia (Supplementary Table S6). We searched for the core taxa in the top 10% of OTUs sorted by maximum abundance: *F. oxysporum* was found in all but one study, *Fusarium* spp. were found in all studies, and *Mortierella* sp. and *Nigrospora* sp. were found in all but two and three, respectively. Every other core genus was identified in at least one study (Supplementary Figure S9). Although dropped from the final core set, *Acromonium* sp. and *Curvularia* sp. were found in seven and five studies of eleven, respectively (Supplementary Figure S10).

## Discussion

To manage *Musa* spp. microbiomes, it is logical to first identify common core taxa as they are the most likely to be present across a range of conditions. Here we focussed on identifying the common core fungal taxa associated with *Musa* spp. To do this we used ITS2 amplicon sequencing to characterise fungal communities in multiple, above, and below-ground compartments of pot and field-grown plants. This was done to assess the entirety of the fungal community associated with the plant. In addition, we sought to consider variation associated with two of the key factors that may differ between farms, *viz.* soil properties and host genotype.

### *Musa* spp. mycobiomes are diverse and differ between plant compartments and soils, but not genotypes

*Compartment*: Plant compartment was the strongest predictor of fungal community diversity and composition, with distinct communities associated with specific tissues. Hence, when comparing results between studies or designing new experiments, it is important to consider the compartment with which the mycobiomes are associated. For example, investigating the mycobiomes of bulk soil is unlikely to yield a result that is representative of fungi associated with roots or other tissues. Our findings also indicate that the fungal communities were diverse in both the ectorhizosphere and leaves, but declined in diversity in the root endophytic compartments. This finding differs from that of a recent investigation of the mycobiome of *Musa* explants and field-grown *Musa* spp. during Fusarium wilt disease progression (Liu et al., 2019). This study found that the endorhizosphere was more diverse than shoots (a pseudostem and rhizome pooled sample). In our study, *Fusarium* spp. were particularly dominant in the root endophytic compartments, whereas in the study of Liu et al. (2019), they were less dominant. As a result, *Fusarium* spp. in our study may have been competitively excluding other fungi in the root endophytic compartment and lowering the overall diversity; similar mechanisms have been demonstrated in other plants (Younginger et al., 2022). Although statistical comparisons of communities were not made by Liu et al., they did report the most dominant taxa being different in various compartments. We too found large differences in community composition between compartments. These distinct communities are likely to arise from compartment-specific ecological niches and environmental conditions as well as the varying ability of the plant to regulate communities in different tissues (Rossmann et al., 2017). Proximal compartments tended to be more similar and leaf communities were distinct from those found in soil-associated compartments. These findings indicate that fungi associated with *Musa* spp. may disperse into endophytic tissues from both the leaves and roots.

*Edaphic factors*: We also observed that the soil in which the plant is grown influences fungal diversity. This is to be expected as it is a major reservoir of fungi available to the plant (Hawksworth and Lu, 2017) and has previously been observed for bacteria associated with *Musa* spp. (Birt et al., 2022). Despite the impact of soil, we found core taxa that were persistently associated with plants grown in different soils. These would be relevant for various farms with differing edaphic conditions. Our investigation revealed differences in both alpha and beta fungal diversity among banana production soils, with larger changes in the root-associated compartments. Nevertheless, interactions between plants and fungi at the root can still have whole plant effects (Chandrasekaran et al., 2019). Differences in fungal diversity have also been observed in studies of banana plantation soils in Spain (Gómez-Lama Cabanás et al., 2021; Ciancio et al., 2022). Within one of these studies, pH was found to differ between soils, as it did in our study (Ciancio et al., 2022). Amendments to pH in banana plantation soils have been shown to influence fungal diversity and may therefore be a driver of fungal diversity in this study (Zhang et al., 2019).

*Host genotype*: Genotype was not found to influence the fungal microbiome of healthy *Musa* spp. in this study. Nevertheless, as some of the cultivars tested are known to be resistant to fungal pathogens (Goldfinger), clearer differences may become apparent when different genotypes experience pathogen pressure. For example, differences in the mycobiome of the leaves of various *Populus* tree genotypes were most apparent when foliar pathogens were present (Cregger et al., 2018). As genotype does not have a large impact on the *Musa* mycobiome, much of the mycobiome research that has been conducted on specific *Musa* genotypes may be translatable to various genotypes in production (Nel et al., 2006b). We also found a similar pattern for bacterial communities in a previous study (Birt et al., 2022). However, an investigation of differences in fungi associated with the seeds of wild *Musa* spp. showed clear differences in the diversity of associated fungal species, with implications for germination rate (Hill et al., 2021). By contrast, our study used sterile tissue culture plantlets (the industry standard) which could have prevented the vertical transmission of fungi.

### The validation of core fungal taxa

The 42 candidate-core fungi microbiome were defined using a pot experiment. We validated our core taxa with a field survey of 52 genotypes because we were aware that there were other genotypes relevant to production that had not been included in the pot experiment, as well as differences between pot and field grown plants (Poorter et al., 2016). Here, we found 21 ‘core’ OTUs, which despite only being 0.35% of OTUs, represented c. 50–60% and c. 35–45% of sequences in roots and above-ground compartments, respectively (Figure 4). All but one of these taxa were also present in all 52 genotypes. In addition, although developmental stage has been shown to influence plant microbiomes (Edwards et al., 2018), these core taxa were found in adult and juvenile plants. Work on other plant mycobiomes have found that dominant fungi often establish early in the plant lifecycle (Johnston-Monje et al., 2021). Given the sterile nature of tissue culture plantlets used in banana production, there may be ample opportunity to introduce core taxa in the plant hardening stage prior to introduction in the field.

### Core OTUs have close banana-associated relatives around the world

To explore a range of climatic zones, different forms of management, and a greater diversity of genotypes and edaphic factors, we checked whether our core taxa could be found in publicly available datasets. We found evidence of the core taxa in Uganda, China, and other parts of Australia. The most dominant of these were the *Fusarium* spp., a *Mortierella* sp., and a *Nigrospora* sp. The consistent association of these fungi could imply they have strong co-evolutionary history (Gross, 2019). Another possibility is that these fungi are environmental generalists and they have been spread through plant matter transported by humans (Golan and Pringle, 2017). This is particularly likely for bananas as they are the world’s most traded fruit and have a long history of being moved around the planet (Voora et al., 2020). Although our data does not allow us to deduce the ecological functions of these core taxa, we could infer their functional importance from network analysis.

### Core OTUs occupy central positions in co-occurrence networks

By combining the dataset from this paper with a previous study of the bacterial microbiome of *Musa* spp. we were able to create a dual amplicon co-occurrence network. Networks such of these can provide increased network stability, higher connectivity, and similar topological re-organization patterns compared single phylogenetic marker networks (Tipton et al., 2018). We found that core fungi occupied central positions within this network (Table 3; Supplementary Figure S5). Fungi with central positions in networks have been shown to have implications for disease incidence in banana production soils (Yang et al., 2022). Interestingly, we also found that core taxa had co-occurrence relationships with *Fusarium* spp., which have important implications for plant health in *Musa* spp. (Ploetz, 2015; Hill et al., 2021).

### Associations of core taxa with host fitness

Circumstantial evidence from the literature also indicates that some of the core taxa are associated with plant health in *Musa* spp. A study into the fungi associated with Fusarium Wilt in *Musa* spp. found that *Cladosporium* spp. were dominant in healthy plants found adjacent to wilting plants, indicating a possible role in disease control (Liu et al., 2019). This study also found *Cladosporium* spp. to persist in tissue culture plants, suggesting a strong association with *Musa* spp. throughout their lifecycle. Moreover, non-pathogenic strains of *Fusarium* associated with *Musa* spp. and have frequently been studied for their ability to control pathogenic *Fusarium* strains (Forsyth et al., 2006; Nel et al., 2006b; Belgrove et al., 2011). Despite being most studied for their ability to cause disease, *F. oxysporum* has been reported to be associated with the rhizosphere of healthy *Musa* spp. (Nel et al., 2006a). Of 60 *F. oxysporum* isolates found by Nel et al. (2006a) to be associated with the rhizosphere of various banana plants, only one isolate caused disease. In total, these isolates could be placed into 12 phylogenetic groups. In a subsequent study, it was demonstrated that some of these *F. oxysporum* isolates were not able to control a pathogenic *F. oxysporum* f. sp. *cubense in vitro* but were highly effective *in planta* (Nel et al., 2006b). The abundance of *Mortierella* spp., another core taxa, has been shown to be positively associated with suppression of Fusarium wilt in *Musa* spp., but decreased in relative abundance the longer a field was under monoculture conditions (Shen et al., 2018). *Mortierella* spp. have been shown to antagonise pathogens through antibiotic production and could perform similar functions for *Musa* spp. (Melo et al., 2014).

Despite being isolated from healthy plants, other close relatives of core fungi have also been associated with causing disease in *Musa* spp.: *Colletotrichum siamense, Nigrospora* sp., and *Cladosporium* spp. are known to cause foliar and post-harvest diseases (Jones, 2000; Surridge et al., 2003; Kumar et al., 2017; Uysal and Kurt, 2020). Nevertheless, non-pathogenic strains of these fungi have been reported. For example, *Colletotrichum* spp. have been isolated from healthy *Musa* spp., but their function was not elucidated (de Lapeyre de Bellaire et al., 2000). Often relatively small changes in fungal genomes can result in pathogenic lifestyle, such as the SIX genes identified in *Fusarium* spp. (Czislowski et al., 2017); without these genes, the fungi may be commensal or mutualist (Van Dam et al., 2017). Nevertheless, taxa may also switch between being mutualist and pathogenic under certain conditions (Slippers and Wingfield, 2007; Ribeiro et al., 2020).

### Comparisons of the core mycobiome of *Musa* with other plant species

The size and composition of the core *Musa* mycobiome has some notable overlaps with other plants. For instance, the core mycobiome of sugarcane (*Saccharum officinarum*) also includes members of the *Cladosporium* and *Pleosporales* (De Souza et al., 2016). Yet, this study found 45 taxa to be core; however, it did not explain core members across different soils which may account for the increased number of taxa considered core. A recent investigation of fungal taxa associated with grapevines (*Vitis vinifera*) explored core taxa across different soil types and found 15 fungal taxa to be core (Liu and Howell, 2021). This investigation also found *Cladosporium* and *Fusarium* OTUs to be core taxa. Similarly, 12 core fungal taxa were found to be associated with the healthy mycobiome of chilli pepper (*Capsicum annuum*) which again included *Cladosporium* and *Fusarium* OTUs (Gao et al., 2021). Together, these results suggest that the core fungi associated with *Musa* spp. may have a broad host range across different plant species.

## Conclusion

This study has given novel insight into the drivers behind the diversity of fungi associated with *Musa* spp. By understanding these, we have defined core taxa consistently associated with *Musa* spp. in various settings. Through network analysis, these taxa have been shown to be more central in community interactions. The function of this set of organisms in conferring health to banana plants is still to be determined. However, this list of core taxa can now provide a focal point for management of these highly complex communities. Future work could investigate whether these taxa result from the style of the production system or an affinity with *Musa* spp. Investigations into their exact functions and how their abundances can be controlled will also provide a better basis for their management use. In the applied use of a core microbiome, there are also opportunities for using these candidates in microbiome manipulation, such as host-mediated microbiome engineering, large-scale bioprospecting, or the introduction of core-microbe consortiums.

## Data availability statement

The datasets presented in this study can be found in online repositories. The names of the repository/repositories and accession number(s) can be found below: https://www.ncbi.nlm.nih.gov/, PRJNA729168.

## Author contributions

PD and AP secured funding and designed the study. HB, PD, and AP collected samples and performed experiments. HB and AR performed marker gene sequencing. HB, PD, and AS analyzed data. HB and PD wrote the paper with input from all authors. All authors contributed to the article and approved the submitted version.

## Conflict of interest

The authors declare that the research was conducted in the absence of any commercial or financial relationships that could be construed as a potential conflict of interest.

## Publisher’s note

All claims expressed in this article are solely those of the authors and do not necessarily represent those of their affiliated organizations, or those of the publisher, the editors and the reviewers. Any product that may be evaluated in this article, or claim that may be made by its manufacturer, is not guaranteed or endorsed by the publisher.
