# Supplementary material for: The core fungal microbiome of banana (Musa spp.)
Source: Front Microbiol. 2023 Mar 30;14:1127779. doi: 10.3389/fmicb.2023.1127779 (PMC10098452; doi:10.3389/fmicb.2023.1127779)
Supplement: Supplementary file 1 [file Data_Sheet_1.PDF]

Supplementary information:

The Core Fungal Microbiome of Banana (*Musa* spp.)

Henry W. G. Birt[1], Anthony B. Pattison[2], Adam Skarshewski[1], Jeff Daniells[2], Anil Raghavendra[1], Paul G. Dennis[1*]

[1]*School of Earth and Environmental Sciences, The University of Queensland, Brisbane, QLD 4072, Australia;* [2]*Department of Agriculture and Fisheries, Centre for Wet Tropics Agriculture, 24 Experimental Station Road, South Johnstone, QLD 4859, Australia;*

*Correspondence: p.dennis@uq.edu.au

Table S1 The abiotic characteristics of five soils used to determine the candidate core fungal microbiome of *Musa* spp. as published previously in Birt et al. (2022).

**Parameter**	**Innisfail**	**Liverpool**	**Pin Gin**	**Tolga**	**Tully**
Latitude (°S)	17.485122	17.454604	17.592257	17.010557	17.481890
Longitude (°E)	145.859047	145.864289	145.833088	145.527065	145.858678
Texture	Clay Loam	Clay Loam	Clay	Clay	Clay loam
Clay (%)	27	26	39	56	28
pH	6.7	7.1	7.1	5.6	6.6
Total C (%)	2.067	1.558	2.417	2.843	2.837
Total N (%)	0.180	0.143	0.212	0.190	0.210
C:N	11.2	10.8	11.5	14.9	13.5

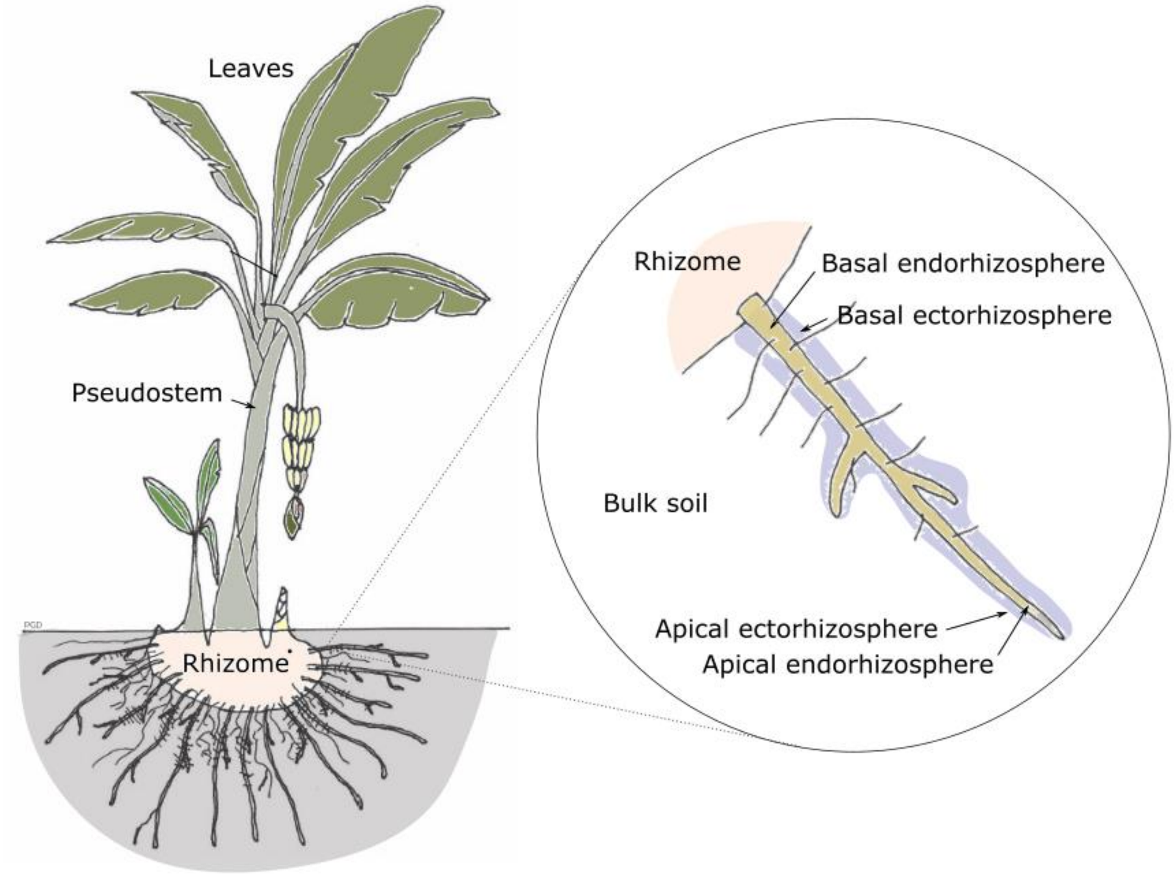

Fig. S1 A diagram showing the various plant compartments sampled in this study.

Table S2 Varieties of *Musa* spp. investigated in the field survey in this study as published in Birt et al. (2022).

Variety	Classification	Genome
845	*Musa acuminata* ssp. *Malaccensis*	AA
846	*Musa acuminata* ssp. *Malaccensis*	AA
848	*Musa acuminata* ssp. *Malaccensis*	AA
850	*Musa acuminata* ssp. *Malaccensis*	AA
851	*Musa acuminata* ssp. *Malaccensis*	AA
852	*Musa acuminata* ssp. *Malaccensis*	AA
Agutay	*Musa acuminata* ssp. *Errans*	AA
Musa balbisiana	*Musa balbisiana*	BB
Blue Java	Ney Mannan	ABB
Bluggoe	Bluggoe	ABB
Borneo	*Musa acuminata* ssp. *Microcarpa*	AA
Calcutta	*Musa acuminata* ssp. *Burmannicoides*	AA
Cam 020	Musa acuminata	AA
Williams	Cavendish	AAA
Ducasse	Pisang Awak	ABB
Dwarf French Plantain	Plantain	AAB
Dwarf Nathan	Cavendish	AAA
FHIA-02	Cavendish hybrid/Pome hybrid	AAAB
FHIA-03	Cooking hybrid	AABB
FHIA-17	Highgate hybrid	AAAA
FHIA-18	Pome hybrid	AAAB
FHIA-23	Highgate hybrid	AAAA
FHIA-25	Cooking hybrid	AAB
GCTCV 218 (Formosana)	Cavendish	AAA
GCTCV 105	Cavendish	AAA
GCTCV 119	Cavendish	AAA
GCTCV 215	Cavendish	AAA
GCTCV 217	Cavendish	AAA
Goldfinger	Pome hybrid	AAAB
Grande Naine	Cavendish	AAA
Gros Michel	Gros Michel	AAA
Highgate	Gros Michel	AAA
Igisahira Gsanzwe	Mutika/Lujugira	AAA
Lakatan	Lakatan	AAA
Lady Finger	Pome	AAB
Ney Poovan	Ney Poovan	AB
Niukin	Pisang Jari Buaya	AA
Pisang Mas	Sucrier	AA
PA 03.22	Pome hybrid	AAAB
Pa Payang	*Musa acuminata* ssp. *Siamea*	AA
Pacific Plantain	Maoli/Popoulu	AAB
Pahang	*Musa acuminata* ssp. *Malaccensis*	AA
Pisang Ceylan	Mysore	AAB
Pisang Gajih Merah	Saba	ABB
Pisang Raja	Pisang Raja	AAB
Red Dacca	Red	AAA
Red Dacca	Red	AAA
Santa Catarina Prata	Pome	AAB
SH-3142	*Musa acuminata*	AA
SH-3142	*Musa acuminata*	AA
SH-3142	*Musa acuminata*	AA
SH-3362	*Musa acuminata*	AA
Sugar	Silk	AAB
Yangambi Km5	Ibota	AAA
Zebrina	*Musa acuminata ssp. Zebrina*	AA

Table S3 The number of replicates in the treatment combinations of soil and plant compartment in plants grown under controlled conditions in this study.

Compartment	Innisfail	Liverpool	Pin Gin	Tolga	Tully
Bulk soil	10	10	10	8	10
Basal ectorhizosphere	10	10	9	10	10
Basal endorhizosphere	10	9	10	9	10
Apical ectorhizosphere	10	10	10	10	10
Apical endorhizosphere	10	9	10	10	9
Rhizome	8	9	4	7	7
Pseudostem	7	10	7	9	7
Leaf	8	6	6	6	5

Table S4 The number of replicates in the treatment combinations of genotype and plant compartment in plants grown under controlled conditions in this study.

Compartment	Cavendish	Gold finger	Lady finger
Bulk soil	10	8	7
Basal ectorhizosphere	10	9	10
Basal endorhizosphere	10	10	10
Apical ectorhizosphere	10	9	10
Apical endorhizosphere	10	10	10
Rhizome	8	6	8
Pseudostem	8	10	5
Leaf	7	8	7

Table S5 The number of replicates for each plant compartment and the total number of genotypes examined in the field survey in this study.

	Bulk soil	Ectorhizosphere	Endorhizosphere	Pseudostem	Leaf
Total number of replicates	164	165	27	26	21
Number of genotypes examined	52	52	18	22	11

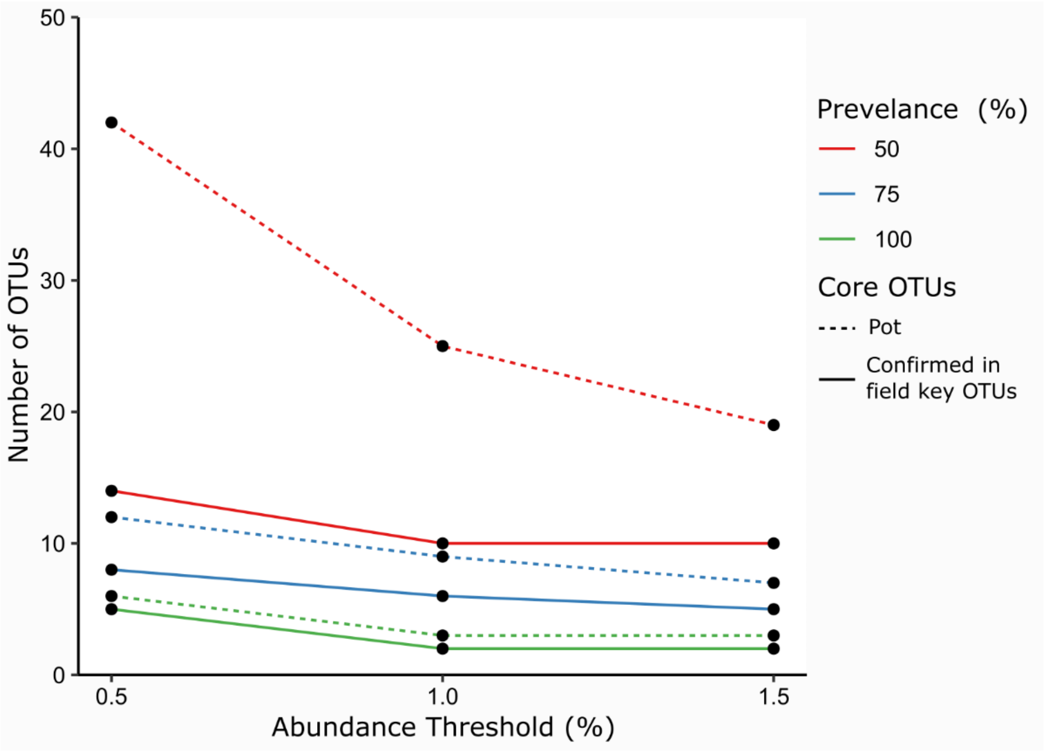

Fig. S2 The impact of different abundance and prevalence thresholds on the number of candidate-core OTUs defined in the pot dataset (dotted line) and confirmed in the key OTUs from the field survey (solid line).

Table S6 Studies included in the meta-analysis in this study.

Study	Method	Plant compartment	Cultivar	Country	Study description	Title	Accession
Fu et al. 2017	Culture independent (454 sequencing of ITS2 region)	Ectorhizosphere	Information not given	China	The effect of a biocontrol on the rhizosphere community of field grown banana plants.	*Inducing the rhizosphere microbiome by biofertilizer application to suppress banana Fusarium wilt disease*	SRP070868
Liu et al. 2019	Culture independent (Illumina MiSeq sequencing of ITS2 region)	Roots and 'shoots'	*Musa* (AAA Group, Cavendish Subgroup) 'Baxi'	China	Examining key microbes present in a plant experiencing Fusarium wilt	*Engineering banana endosphere microbiome to improve Fusarium wilt resistance in banana*	SRP061527
Rames et al. 2018	Culture independent (Illumina MiSeq sequencing of ITS2 region)	Bulk soil	*Musa* (ABB Group, Pisang Awak Subgroup) 'Ducasse'	Australia	Examining the effect of ground cover on the microbiome of banana	*Soil microbial community changes associated with ground cover management in cultivation of Ducasse banana (Musa sp. ABB,*	PRJNA397084

						Pisang Awak subgroup) and suppression of Fusarium oxysporum	
Rossman et al 2012	Culture dependent	Bulk soil, ectorhizosphere, pseudostem exterior	Information not given	Uganda	Examination of the soil and pseudostem of field-grown bananas.	*Banana-associated microbial communities in Uganda are highly diverse but dominated by Enterobacteriaceae*	HE586686 to HE586734
Shen et al. 2015a	Culture independent (454 sequencing of ITS2 region)	Bulk soil	*Musa* (AAA Group, Cavendish Subgroup) 'Brazil'	China	Comparing fields of where a biocontrol has been applied with one where it has not	*Rhizosphere microbial community manipulated by 2 years of consecutive biofertilizer application associated with banana Fusarium wilt disease suppression*	DRA002434

Shen et al. 2015b	Culture independent (Illumina MiSeq sequencing of ITS2 region)	Bulk soil	*Musa* (AAA Group, Cavendish Subgroup) 'Brazil'	China	Introduction of biofertiliser to control Fusarium wilt in pots.	*Effect of biofertilizer for suppressing Fusarium wilt disease of banana as well as enhancing microbial and chemical properties of soil under greenhouse trial*	DRA002820
Shen et al. 2018a	Culture independent (Illumina MiSeq sequencing of ITS2 region)	Bulk Soil	*Musa* (AAA Group, Cavendish Subgroup) 'Brazil'	China	The effect of soil fumigation on the soil microbiome	*Soil pre-fumigation could effectively improve the disease suppressiveness of biofertilizer to banana Fusarium wilt disease by reshaping the soil microbiome*	SRP112719
Shen et al. 2018b	Culture independent (Illumina MiSeq sequencing of ITS2 region)	Bulk soil	*Musa* (AAA Group, Cavendish	China	Effect of monoculture span on the soil microbiome	*Banana fusarium wilt disease incidence is influenced by shifts of soil microbial*	DRA004913

			Subgroup) 'Brazil'			*communities under different monoculture spans*	
Shen et al. 2019	Culture independent (Illumina HiSeq sequencing of ITS1 region)	Bulk soil	*Musa* (AAA Group, Cavendish Subgroup) 'Brazil'	China	Soil samples from pot experiment after fumigation and the addition of a biocontrol to control Fusarium wilt.	*Suppression of banana Panama disease induced by soil microbiome reconstruction through an integrated agricultural strategy*	SRP111447
Wang et al. 2015	Culture independent (454 sequencing of ITS2 region)	Bulk soil	Information not given	China	The effect of crop rotation on the soil microbiome	*Pineapple-banana rotation reduced the amount of Fusarium oxysporum more than maize-banana rotation mainly through modulating fungal communities*	DRA002472

Zhang et al. 2019	Culture independent (Illumina HiSeq sequencing of 18S region)	Bulk soil	*Musa* (AAA Group, Cavendish Subgroup) 'Williams'	China	The effect of liming and organic fertiliser on the soil microbiome	*Organic fertilizer, but not heavy liming, enhances banana biomass, increases soil organic carbon and modifies soil microbiota*	SRP139145

Table S7 The impact of soil, genotype and plant compartment on alpha diversity metrics assessed by ANOVA from the fungal microbiome of *Musa* spp. These results derive from our pot experiment which included five distinct soils, three *Musa* spp. genotypes, and eight compartments.

**Response variable**	**Predictor variable**	***F* value**	***P* value**
Observed OTUs	Compartment	23.4	<0.001 ***
	Soil	1.1	0.347
	Compartment: Soil	1.0	0.503
Predicted OTUs (Chao 1)	Compartment	16.3	<0.001 ***
	Soil	1.6	0.162
	Compartment: Soil	0.9	0.622
Observed OTUs	Compartment	16.2	<0.001 ***
	Genotype	1.1	0.320
	Compartment: Genotype	1.1	0.389
Predicted OTUs (Chao 1)	Compartment	12.5	<0.001 ***
	Genotype	1.9	0.159
	Compartment: Genotype	1.2	0.277

Table S8 Average percentage similarity plus/minus the standard deviation of fungal community composition in various *Musa* spp. plant compartments. Percentages were produced using a Bayesian approach implemented through SourceTracker.

	Source							
**Sink**	**BS**	**AER**	**AEnR**	**BER**	**BEnR**	**R**	**PS**	**L**
**BS**	-	94.3 ± 1	75.6 ± 13	95.9 ± 1	77.3 ± 9	67.1 ± 11	63.4 ± 11	61.1 ± 11
**AER**	94.6 ± 1	-	81.0 ± 8	96.1 ± 1	80.1 ± 6	71.1 ± 5	63.2 ± 10	59.7 ± 12
**AEnR**	93.8 ± 6	95.1 ± 6	-	94.9 ± 6	92.9 ± 4	89.8 ± 5	83.7 ± 8	83.2 ± 8
**BER**	94.5 ± 2	94.9 ± 1	76.3 ± 11	-	78.3 ± 10	65.1 ± 13	57.6 ± 9	68.1 ± 9
**BEnR**	78.8 ± 6	80.0 ± 5	79.4 ± 9	79.8 ± 5	-	80.2 ± 9	67.9 ± 12	55.8 ± 11
**R**	74.1 ± 2	71.9 ± 9	75.0 ± 13	75.6 ± 4	83.5 ± 10	-	79.6 ± 13	76.7 ± 13
**PS**	61.9 ± 6	62.3 ± 8	62.3 ± 18	63.4 ± 10	87.6 ± 7	91.0 ± 3	-	91.3 ± 2
**L**	40.1 ± 11	53.5 ± 8	44.1 ± 18	43.7 ± 14	55.5 ± 12	66.7 ± 9	63.3 ± 15	-

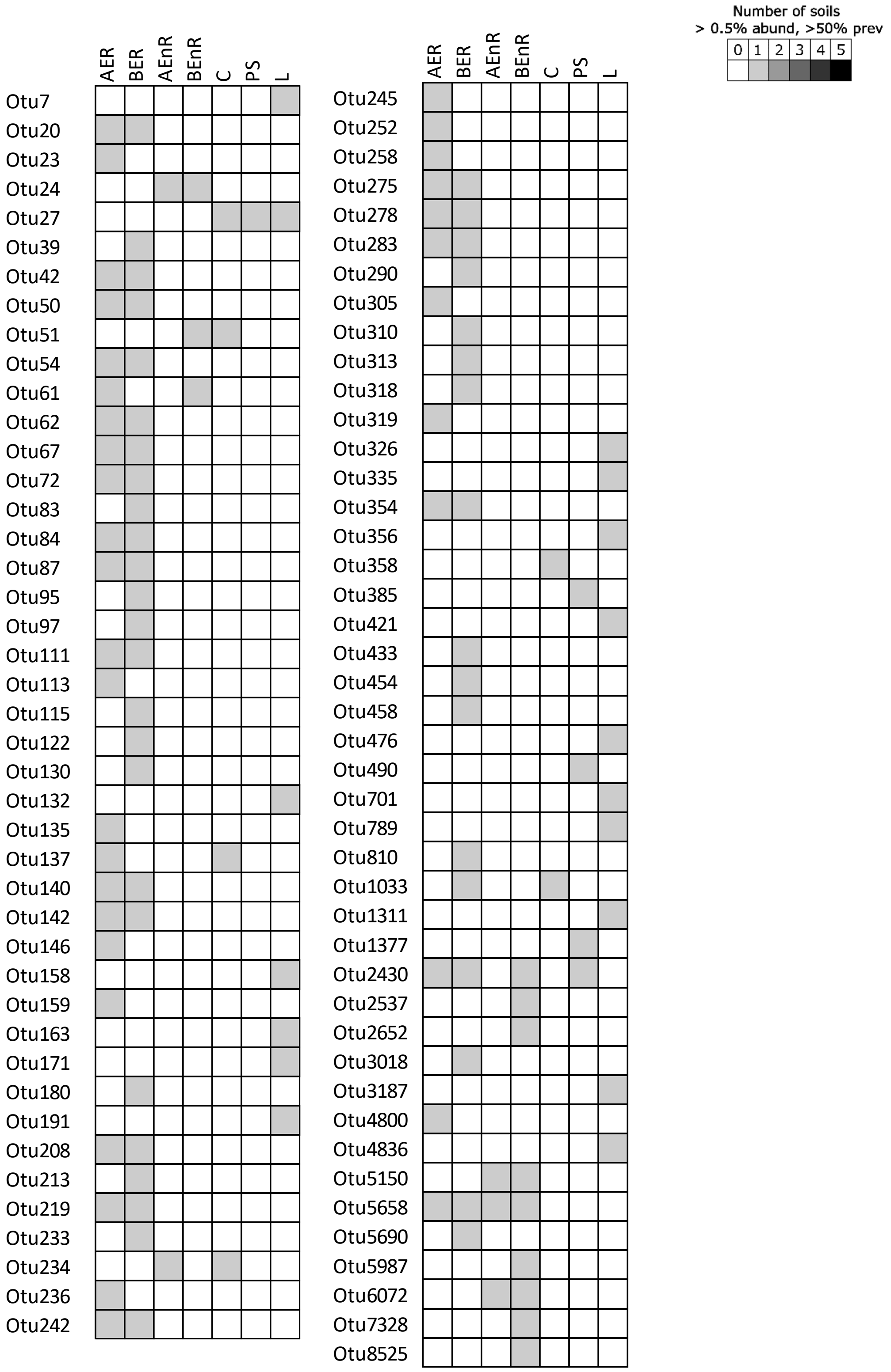

Fig. S3 Heatmaps to show the number of OTUs identified as abundant and prevelant in plant compartments of *Musa* (AAA Group, Cavendish Subgroup) 'Williams' grown in a single soil

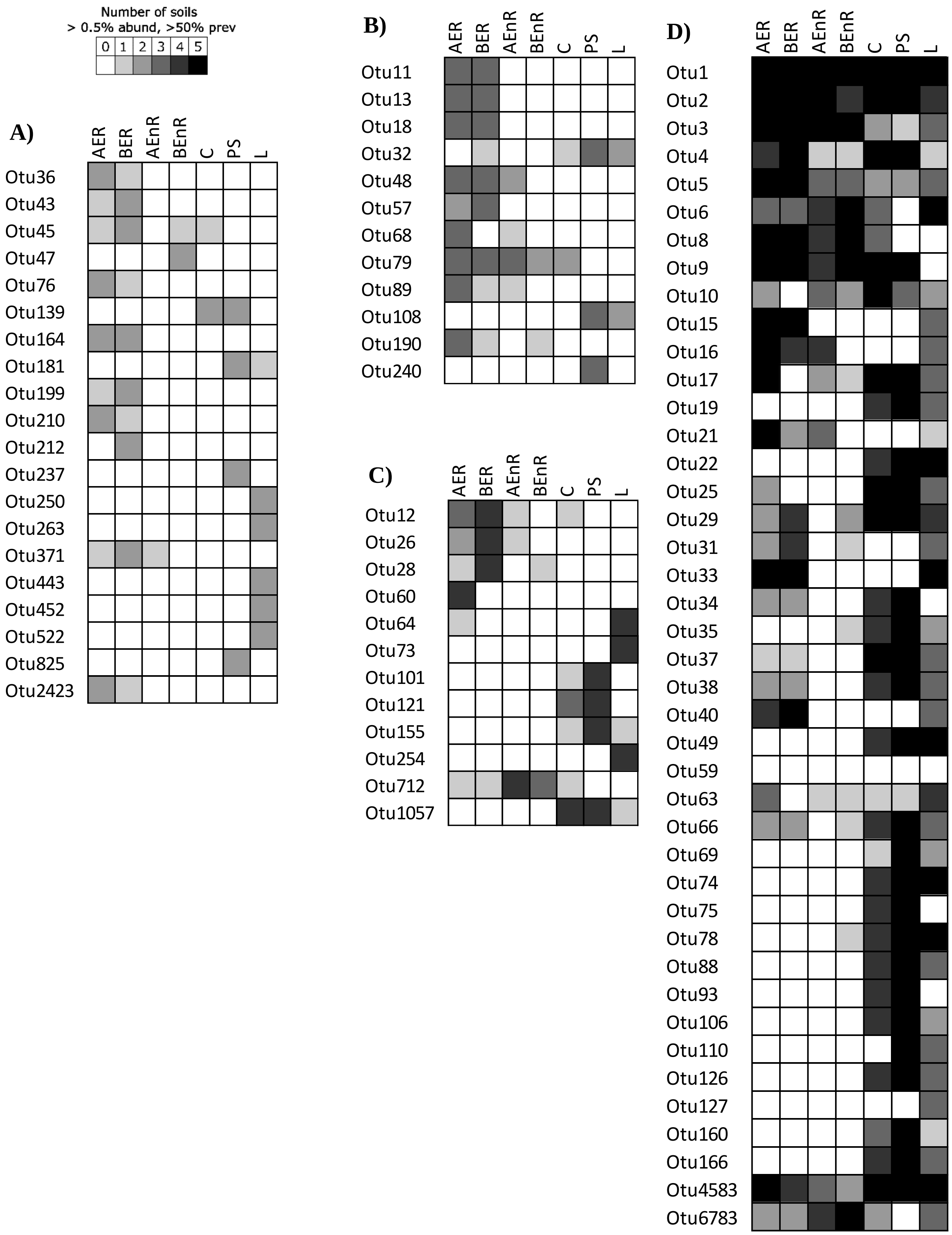

Fig. S4 The OTUs identified as abundant and prevalent in plant compartments of *Musa* (AAA Group, Cavendish Subgroup) ‘Williams’ grown in two (A), three (B), four (C), and five (D) soils

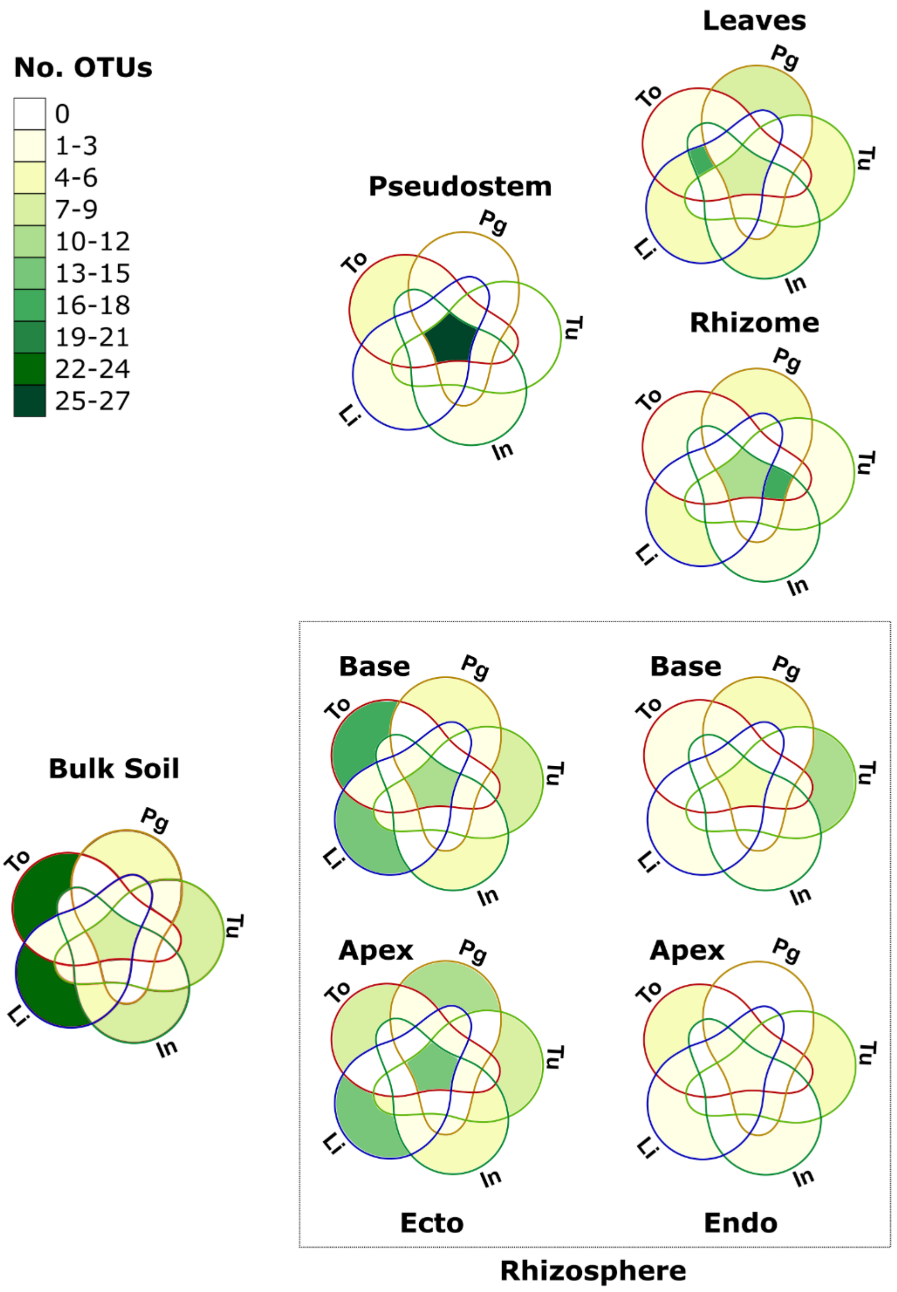

Fig. S5 Venn diagrams showing the number of OTUs found at an average abundance > 0.5% and prevelance > 50% in *Musa* (AAA Group, Cavendish Subgroup) 'Williams' plants grown in five distinct soils.

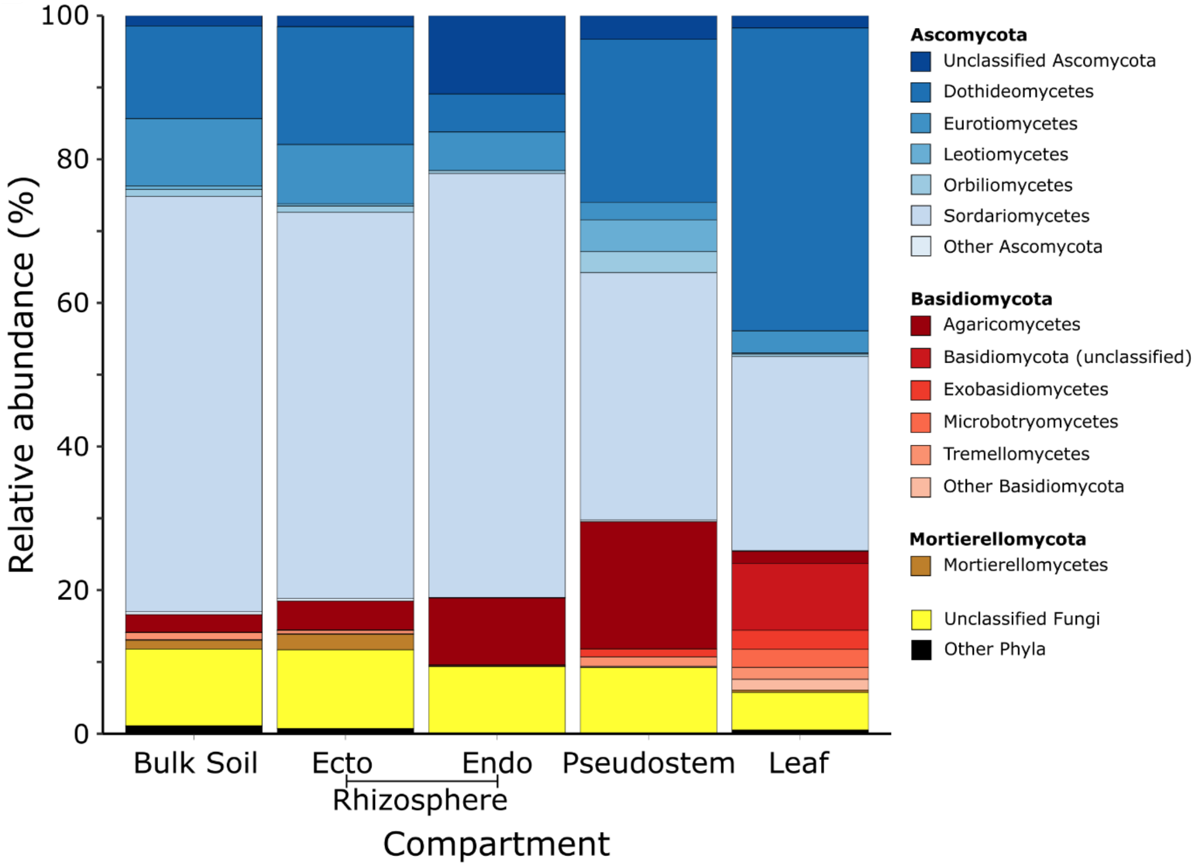

Fig. S6 The mean relative frequencies of fungal classes in different plant compartments associated with various field grown *Musa* genotypes. Within each phylum, classes represented at <1% mean relative abundance are grouped as other. Abbreviations are as follows: endo – endorhizosphere, ecto – ectorhizosphere.

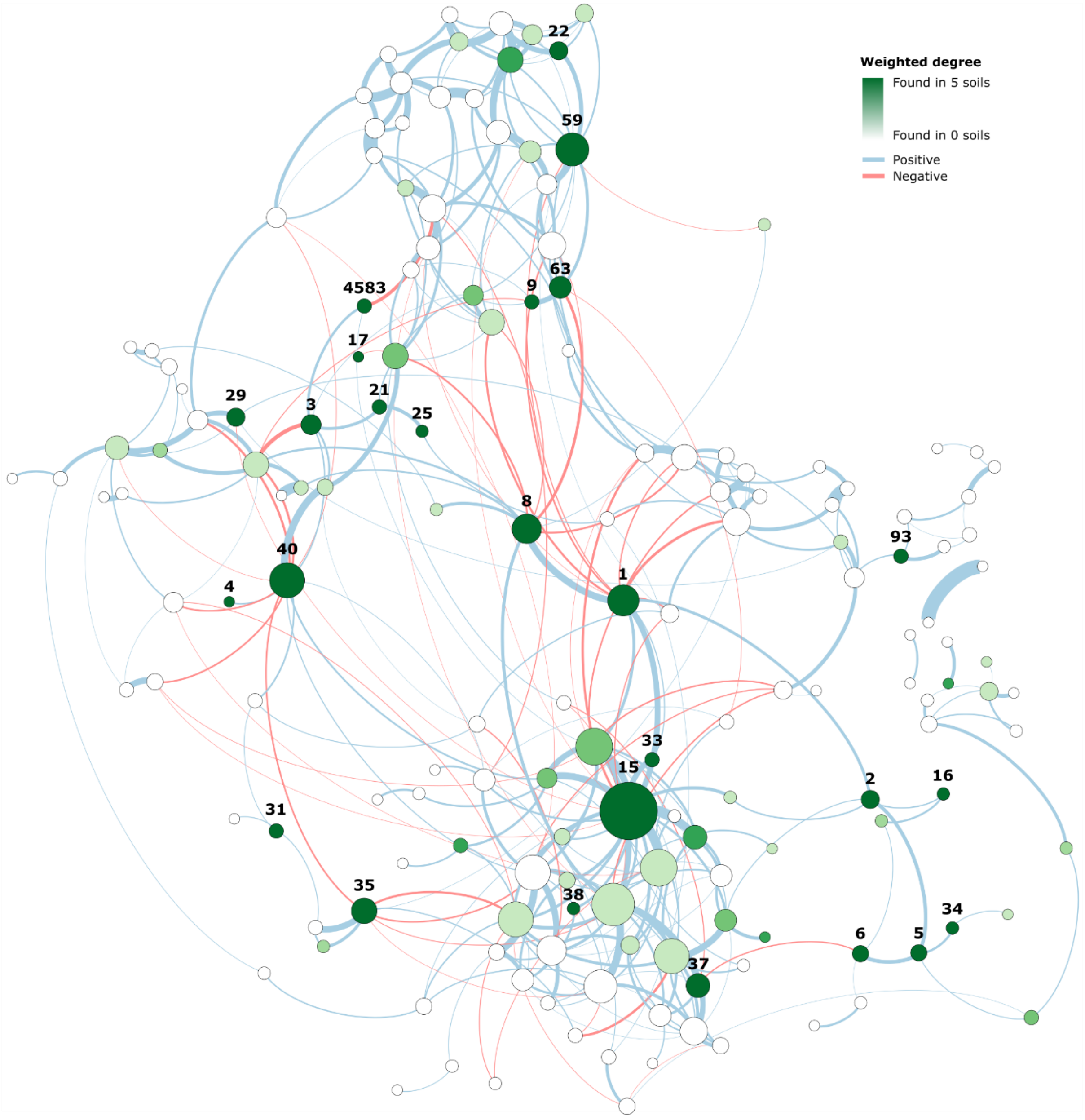

Fig. S7 A network showing co-occurrence between fungal OTUs associated with adult *Musa* spp. in a field setting. Node size is proportional to node degree. Nodes are coloured by the number of soils each OTU was present in according to a previous pot experiment.

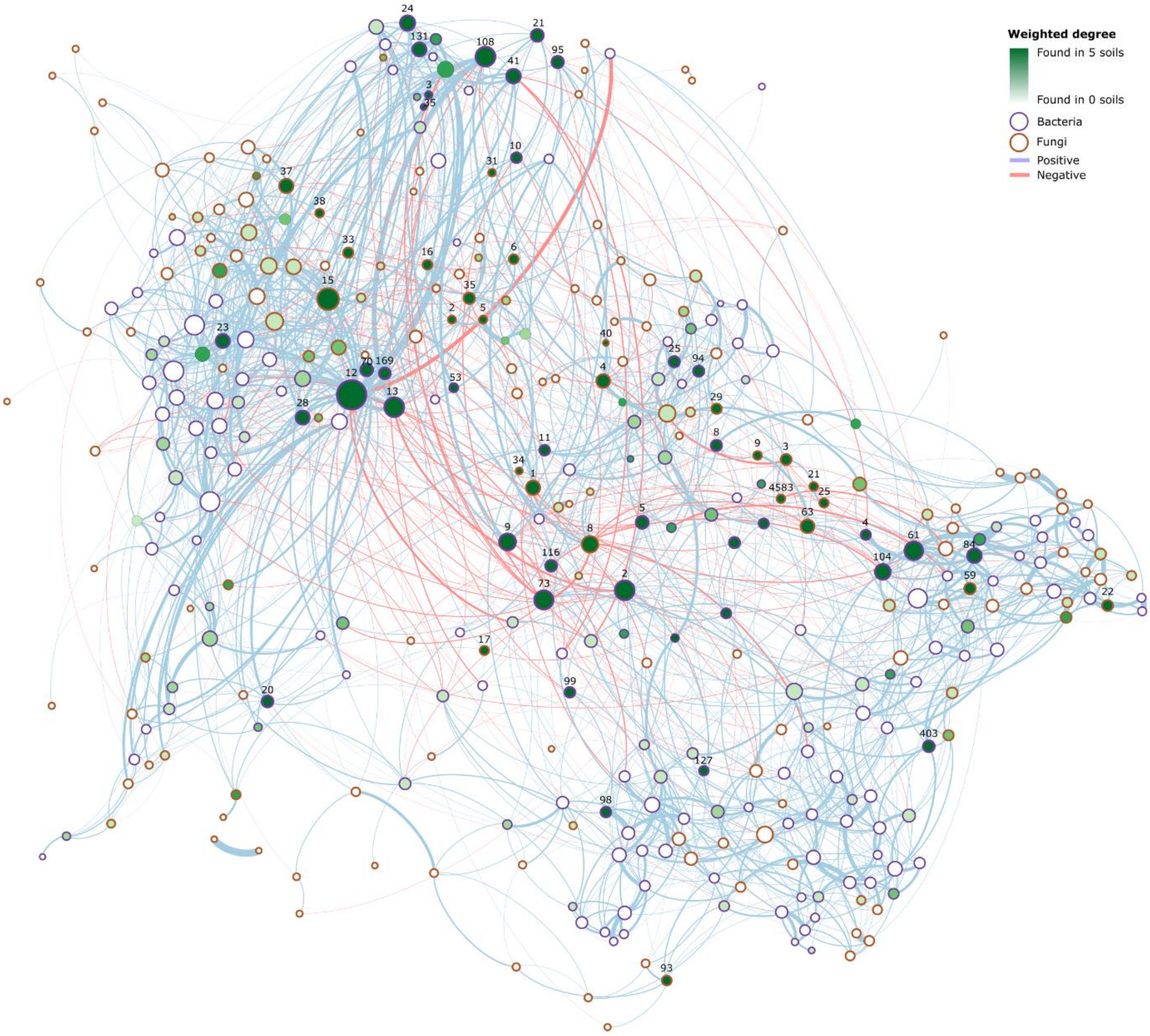

Fig. S8 A dual amplicon (16S and ITS2) network showing co-occurrence between fungal and bacterial OTUs associated with 52 adult *Musa* spp. in a field setting. Node size is proportional to node degree.

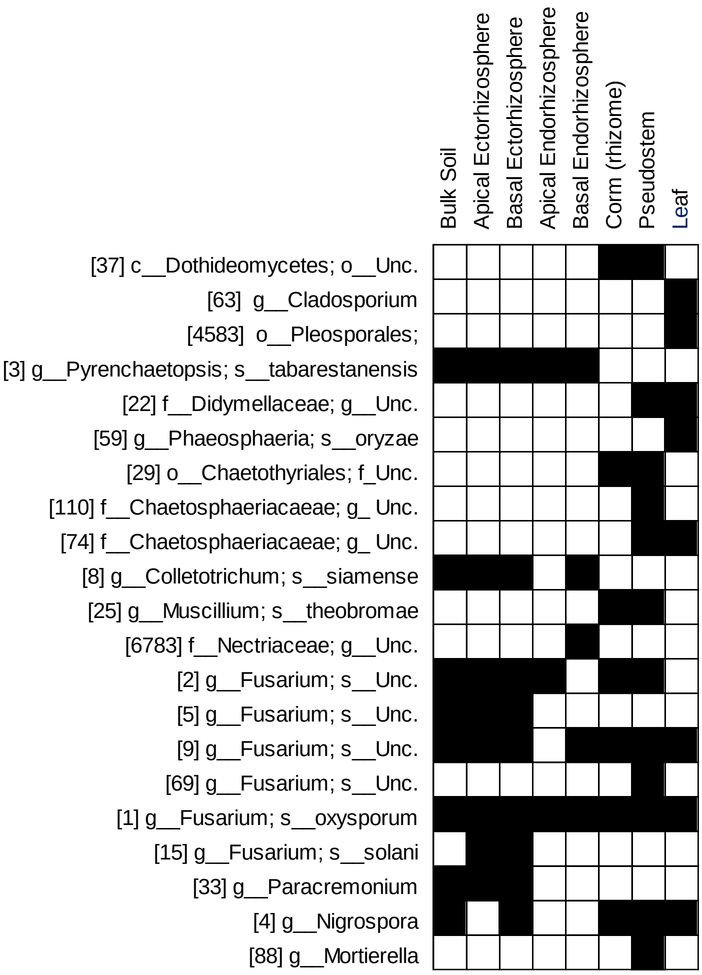

Fig. S9 The final core *Musa* spp. fungal microbiome. Black tiles indicate which plant compartments each OTU was identified as core.

Table S9 Representative sequences the 42 candidate-core OTUs found in all soils from *Musa* spp. grown under controlled conditions. OTUs that were elevated to full core-status are marked as core in bold text.

OTU	Sequence (5'-3')
**Otu1**	CAACCCTCAAGCACAGCTTGGTGTTGGGACTCGCGTTAATTCGCGTTCCTCAAATTGATTGGCG
**Core**	GTCACGTCGAGCTTCCATAGCGTAGTAGTAAAACCCTCGTTACTGGTAATCGTCGCGGCCACGC
	CGTTAAACCCCAACTTCTGAA
**Otu2**	CAACCCTCAGGCCCCCGGGCCTGGCGTTGGGGATCGGCGGAAAGCCCCCTGTGGGCATACGC
**Core**	CGTCCCCTAAATACAGTGGCGGTCCCGCCGCAGCTTCCATTGCGTAGTAGCTAACACCTCGCAA
	CTGGAGAGCGGCGCGGCCAAGCCGTAAAACCCCCAACTTCTGAA
**Otu3**	TGTACCCTCAAGCACTGCTTGGTGTTGGGCGTTTGTCCTGCAAAGGACTCGCCTGAAAGCGATT
**Core**	GGCGGCCAACGTACTGGTGGTAGAGCGCAGCACAATTTGCGTCTCTCCCTTCTACGTCGGCGT
	CCATGAAGCCTTTTTTCAAC
**Otu4**	CAACCCCTAAGCACAGCTTATTGTTGGGAATCTACGCCCTAGTAGTTCCTCAAAGACATTGGCGG
**Core**	AGTGGCAGTAGTCCTCTGAGCGTAGTAATTCTTTATCTCGCTTTTGTTAGGTGCTGCCTCCCCGG
	CCGTAAAACCCCCAATTTTTTCT
**Otu5**	CATCCCTCAAGCCCCAGCGGCTTGGTGTTGGGCTTCGGCCGTCCTCAGCGGCGGCCGTGCCCC
**Core**	AAATACAGTGGCGGTCTCGCCCCCGGCTCCTCTGCGTAGTAGTAACATCTCGCACTGGGACGG
	AGCGAAGGCCACGCCGTAAAACAACCCAACTTTCTGAA
Otu6	CAACCCTCAAGCCCCCGGGCTTGGTGTTGGGGATCGGCGAGCCTCTGCGCCCGCCGTCCCCTA
	AATTGAGTGGCGGTCACGTTGTAACTTCCTCTGCGTAGTAGCACACTTAGCACTGGGAAACAGC
	GCGGCCACGCCGTAAAACCCCCAACTTTTGAAC
**Otu8**	CAACCCTCAAGCTCTGCTTGGTGTTGGGGTACTACAGCCTCCTGTAGACCCTGTAACGTAGTGG
**Core**	CGGATCCTCTGTAAACCTGAGCGTAGTAGTTACTTCTCGCTTCTGGCAAGCAGCGGTTTCCACG
	CCGTGAAACCCATACTTTTAT
**Otu9**	CAACCCTCAAGCTCAGCTTGGTGTTGGGACTCGCGGTAACCCGCGTTCCCCAAATCGATTGGCG
**Core**	GTCACGTCGAGCTTCCATAGCGTAGTAATCATACACCTCGTTACTGGTAATCGTCGCGGCCACG
	CCGTAAAACCCCAACTTCTGAA
Otu10	CAACCCTCAAGCCTAGCTTGGTGTTGGGCGTTTGTCCCGCCTCCGCGCGCCTGGACTCGCCTC
	AAAAACATTGGCGGCCGGTTCCCAGCAGGCCACGAGCGCAGCACAGCGAGCGCTGAAGTGGCT
	GCGGGTCGGCGCACCAGAAGCCCCCCCACACCAGAA

Otu15 CAACCCTCAGGCCCCCGGGCCTGGCGTTGGGGATCGGCGGAAGCCCCCTGCGGGCACAACGC
Core CGTCCCCCAAATACAGTGGCGGTCCCGCCGCAGCTTCCATTGCGTAGTAGCTAACACCTCGCAA
CTGGAGAGCGGCGCGGCCACGCCGTAAAACACCCAACTTCTGAA

Otu16 CAACCCTCGAGCCCTCGTGGCCCGGCGTTGGGGATCTGCCACGGCAGGCCCCGAAATACAGTG
GCGGACCCGTTAGGCCCTTCCTTTGCGTAGTAGCATTAGCCTCGCATCGGGAGCCGGCGGGCT
CTCCTGCCTCTAAACCCCCCAACAAGCCCGCTCCGGCGGCACCAA

Otu17 CAACCCTCGAGCCCCCGTGGCCCGGCGTTGGGGATCTGCCCAGGCAGGCCCCTAAAACCAGT
GGCGGACCCGTTACAGGCCCTTCCTTTGCGTAGTAGCATCAGCCTCGCATCGGGAGCCAGCGG
GCTTCTGGCCTCTAAACCCCCATCAAGTCCGCCCCGGCGGCACCAA

Otu19 ACACCCTCAAGCTCTGCTTGGTGTTGGGCGTCTGTCCCGCCTCCGTGCGCGGACTCGCCTCAA
AGTCATTGGCAGCGGTCTCGTCGGCTTCTCGCGCAGCACATTTGCGCTTCTCGGAGCCCCGGC
GGATCAGCGTCCAGCAAGCAATTTCATG

Otu21 CAACCCTCGAGCCCTCGTGGCCCGGCGTTGGGGATCTGCCCAGGCAGGCCCCGAAAACCAGT
GGCGGACCCGTCCGGGACCTCTCCTTGCGTAGTAGCATCAGCCTCGCATCGGGAGCCGGCGG
GCCTTCCGGCCTCTAAACCCCCCACAAGTCCGCTCCGGCGGCATCAA

Otu22 GTACCTTCAAGCTTTGCTTGGTGTTGGGTGTTTGTCTCGCCTCTGCGTGTAGACTCGCCTTAAAA
Core CAATTGGCAGCCGGCGTATTGATTTCGGAGCGCAGTACATCTCGCGCTTTGCACTCATAACGAC
GACGTCCAAAAGTACATTTTTACAC

Otu25 CAACCCTCGAGCCCCCGTGGCCCGGCGTTGGGGCCCTACGCGCCTTCTGGCAGTAGGCCCCG
Core AAAAGCAGTGGCGGTCCCGTGTGGTCCTCTCCTTTGCGTAGTAGCATTAGCCTCGCTTTGGGAG
CCCGCGGCGTGCCGGCCTCGAAACCATCAAGTCCGCTCCGGCGGCACCCA

Otu29 TCACCCCTCAAGCCCGGCTTGTTGTTGGACGCCGGCGGTGGCCTCTTTTGGCCCCGCCCGTCT
Core CAAAGATAATGACGGCGTCTGTGAGGACTCCTGTACACTGAGCTTTCGGGCACGTACTAGGCAG
CACTTCAGGCCCGGTCTTCGTCGAATCCCATCTCGGGTGCCGACAAACTTTTACCAA

Otu31 GTACCCTCAAGCTTTGCTTGGTGTTGGGCGTTTTTTGTCTTTGGTTTTGTCCAAAGACTCGCCTTA
AAACGATTGGCAGCCGGCCTACTGGTTTCGCAGCGCAGCACATTTTTGCGCTTGCAATCAGCAA
AAGAGGACGGCACTCCATCAAGACTCTATATCAC

Otu33 CAACCCTCAAGCCCCCTCGGGGGATTGGTGCTGGGGATCGGCCGTCATGGGCCGGCCCCGAA
Core ATCGAGTGGCGGTCTCGCCGTCGCCTCCTCTGCGTAGTAGAAACACCTCGCACTGGGACGCGG
CGCGGCCATGCCGTTAAACCCCCGACTTCTGAAA

Otu34 CAACCCTCAAGCCCCCCGGGCTTGGTGTTGGGGATCGGCACAAGGCCCCTCGCGGCCCGCCG
TCCCCCAAATGCAGTGGCGGTCACGTCGCAGCCTTCTATGCGTAGTAGCAACACCTCGCACTGG
AGCGCGACGCGGCCACGCCGTAAAACCCCCGACTTTTTTCT

Otu35 AGACTCAATCCCTCGGGTTTCCGAGGAGATTGGACTTGGGTGTTGCCGCTCTGCCGGCTCGCCT
TAAAAGACTTAGCGGGATAGCACCGTAGTCGGCGTAATAAGTTTCGTCGGTGAAGGTTGTGATG
ACTGCTTACAATCGCCCTCGGGCAATTTTTGA

Otu37 ACACCCCTCAAGCTCTGCTTGGTGTTGGGCGTCTGTCCGCCGCCGCGAGGCGGGGGACTCGC
Core CCCAAAGGCATTGGCAGCGGCGTCCGCCGGGCTTTTCCCCATCAGCGCAGCACTGTTCGCGCA
CCTCGGGGACCCGCGCGGAGGCAGCGTCCACGAAGCGCCCACGTTTTTG

Otu38 CGACCCTCGCGCCCGGCTTCTGTCGGGGGCGGTGTTGGGGATCGGCCACACCCTTCAGTGGG
AGGCCGGCCCCTAAATTCAGTGGCGACCACGCTGTAGCCTCCCCTGCGTAGTACTAAAACCACC
TCGCAGGCGGAGAGCGGTGCGGCCCGCCGTAAAACCCCCCAACTTTTACAA

Otu40 CAACCCCTAAGCACAGCTTATTGTTGGGCGTCTACGTCTGTAGTGCCTCAAAGACATTGGCGGA
GCGGCAGCAGTCCTCTGAGCGTAGTAATTCTTTATCTCGCTTCTGTTAGGCGCTGCCCCCCCGG
CCGTAAAACCCCCAATTTTTTCT

Otu49 GAACCCTCAAGCTCTGCTTGGTGTTGGGTGTTTGTCCCGCCATTGCGCGTGGACTCGCCTTAAA
GTAATTGGCAGCCATGTAATTCGGCTTTGAGCGCAGCACATTGCGTACTCTATGCTGGTACATTG
GCATCCAGAAGCCCTTTTTTAC

Otu59 GTACCTTCAAGCTTTGCTTGGTGTTGGGTGCTTGTCTTTTTGTTAAGACTCACCTCAAAGTCATTG
Core GCAGCCAGTGTTTTGGTAGTAAGCGCAGCACATTTTGCGTCTTGGTCCCTTAACAGCAGCATCC
ATCAAGCCATTTTCTCAC

Otu63 CACCACTCAAGCCTCGCTTGGTATTGGGCAACGCGGTCCGCCGCGTGCCTCAAATCGACCGGC
Core TGGGTCTTCTGTCCCCTAAGCGTTGTGGAAACTATTCGCTAAAGGGTGTTCGGGAGGCTACGCC
GTAAAACAACCCCATTTCTAA

Otu66 CATCCCTCAAGCCCCTTCGGGCTTGGTGTTGGGCATCGGCCGTCCCTCCAGCGGCGGCCGTGC
CCCAAATACAGTGGCGGTCTCGCCCCCGGCTCCTCTGCGTAGTAGTAACATCTCGCACTGGGAC
GGAGCGTAGGCCACGCCGTAAAACAACCCAACTTTCTGAA

Otu69 CAACCCTCAGGCCCTGCCTGGTGTTGGAGGACTGCGCACCGCAGCCTCCCAAAGCAAGCGGCG
Core GCGGCGCCCCATAACCGAACGCAGTAGTTACATCTCGTTCTGGGTCTGGGTCGTTGTCCTGCCG
GAAAACCCCCATCTTTTTAAT

Otu74 ACACCCCTCAAGCTCTGCTTGGTGTTGGGCGTCTGTCCGCCGCCGCGAGGCGGGAGGACTCGC
Core CCCAAAGGCATTGGCAGCGGCGTCCGCCGGGCTTTCCCCATCAGCGCAGCACTGTTCGCGCAC
CAACGGGGACCCGCGCGGAGGCAGCGTCCAGGAAGCGCCCACTTTTTTG

Otu75 CAACCCTCAAGCTCTGCTTGGTATTGGGCCCTGCCGCTGGGCAGGCCTTAAAATCAGTGGCGGT
GCCGTCGCGGCTCCAAGCGTAGTACATCTCTCGCTCCGGAGGCCCGGTGGTGTCTCGCCAGAC
AACCCCCAAATTGTCTTCTAT

Otu78 CATCATCATCTCCTCCCCGGTTTTGCCGGGTTCGGAAGGTGGACTTGGAGGACTTTTGCTGCTG

CACAAGCGGCTCCTCTTGAATGCATCGGCTGTGCTCGAAAGGGCGCTCCTTGACGTTGTAATCT

TCCTCGTCTCGGGCTTCTTTCTGACCGGCCTTGGCCTTTGCCTCTGGAGTGGCGGCCAACTTTT

TACTTTGACATCTGACCTCGAATCGGG

Otu88 AACACCTCAAAGCTTTTGATCTTTTTCAAAAGTTTTGGACTTGAGCAATCCCAACACCAGTCTTTT

Core TGGATCGGTGGCGGGTTGCTTGAAATGCAGGTGCAGCTGGACATTCTCCTGAGCTAAAAGCATA

TTTATTTAGTCCCGTCAAACGGATTATTACTTTTGCTGCAGCTAATATAAAGGGAGTTTGACCGTT

TTGGCTGACTGATGCAGGATTT

Otu93 CAACCCCTCGGACCCCCCGCGGCCCGGCGTTGGGGACCTGCCACGGCAGGCCCCGAAAACCA

GCGGCGGACCCTCCGGGCCCTCTCCTTGCGCAGTAGCATCAGCCTCGCATCGGGAGCCCGGG

CGGCGTCCGGCCCCTAAACACCCCCAAAGCCCGCCCCCGCGCGGCACCAA

Otu106 AAACCCTCAAGCCCCATGGGTGTTGTCCCGCCTCGTGCGCGGCCTTCGCCTGGCTTGGTGTTG

GGTGCCTGTCCCTGCTCCCCGCGGGACTCACCCCAAATGCATTGGCAGCAGCCCCTCGGCTTC

CCGCGCAGCACATTGCGCAGCGGAGCGTAGAGAGGCGTGCGTCCAGCAAGCAAACCCCAA

Otu110 CAACCCTCAGGCCCTGCCTGGCGTTGGAGGACTGCGCACGCAGCCTCCCAAAGCGAGCGGCG

Core GCGGCGCCCCGAAACCGAACGCAGTAGTTACTTCTCGTTCTGGGTCCGGGTCGTTGTCCTGCC

GGAAAACCCCCTTCTAATCAAT

Otu126 CAACCCTCGAACCCCGGTTTTCTTTATGGATCCCGGGGATCGGTGTTGGGGCACTACGGAGGTC

TTCTGACCGCCGTAGGCTTCGAAATACAGTGGCGGTCCCGCCGCGGCGCCCTCTGCGTAGTAA

TTTTCACCTCGCATTGGGTCCCGGCGAAGGCCAGCCGTCAAACCCTCTATTTTCTATG

Otu127 TGTACCCTCAAGCACTGCTTGGTGTTGGGCGTTTGTCCTGCAGAGGACTCGCCTGAAAGCGATT

GGCGGCCAACGTAGTCGTGGCAGAGCGCAGCACAATCTCGCGTCTCTCCCCTCTGCGTCGGCG

TCCATAAGCCTACAATCTCAAC

Otu160 ACACGATCAACTCTAGTTAGAGTTGGGATTGGCCTCATGCACTGTTATAGTGTATCTGGCTCGAA

TGGATCAAGTTGATGGGCTCCCCAGCGATGCTCGATGTGTGCTAGCGAGACAGTCGTCCCGATC

TCACATTAGTGGTTTTGTTGCAGAGCTCTAATCACAGCTTGTTAACACATATACAAATCTA

Otu166 AACAAACCCATCGGGTGCTGGGATCCACGGATCCCTCACCCGGTTCTGAAGGTGGGATGCACG

CAATCCCTCTTTGAAAGGAATCGGCGATGCTTGCTTGTCCATTTGGTTGCGTAGTAAAATTACCT

TTACCTGTCGCAGTTGGACAAGCAATGATCTGCCATAAAACCGTAGAGCCTGCATGGCTTCAGT

Otu4583 TGTACCCTTAAGCACTGCTTGGTGTTGGGCGTTTGCCCTGCAAAGGACTCGCCTGAAAGCGATT

Core GGCGGCCAACGTACTGGTGGTAGAGTGCAGCACAATTTGCGTCTCTCCCCTCTGCGTCGGCGT

CCATGAAGCCTTTTTTCAAC

**Otu6783**	CAACCCTCAAGCCCCTGGGCTTGGTGTTGGGGATCGGCGAGCCTCTGCGCCCGCCGTCCCCTA
**Core**	AATTTAGTGGCGGTCACGTTGTAACTTCCTCTGCGTAGTAGCACACTTCGCACTGGGAAACAGC
	GCGGCCACGCCGTAAACACCCCAACTTTTAAAC

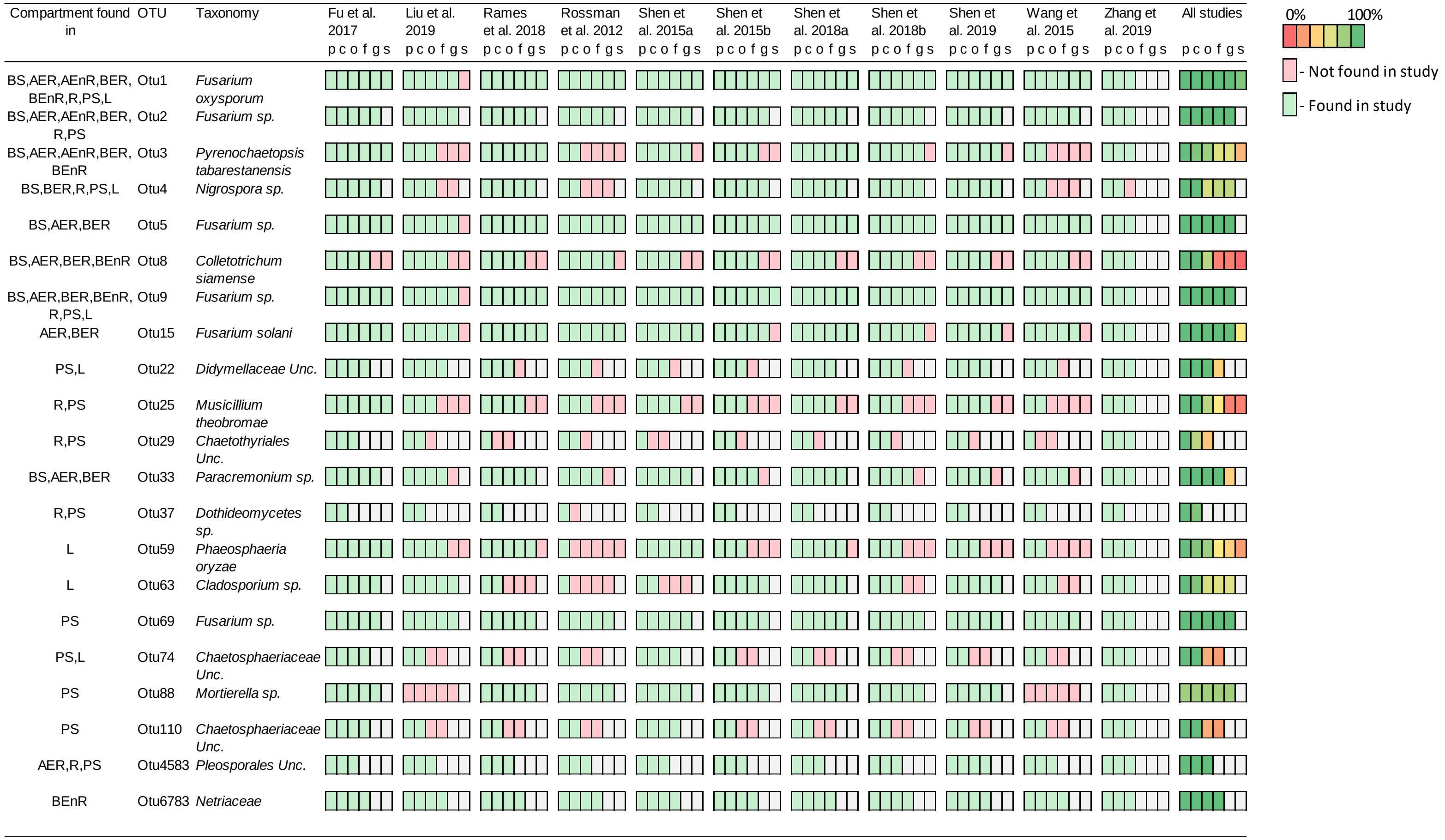

Fig S10 The presence of core OTUs in the taxonomy assigned to the top 10% of OTUs by maximum abundance in publicly available datasets that examine fungi associated with *Musa* spp. Green tiles represent presence of an OTU in a study with the same taxonomy as a core OTU, red indicates they were not present, white indicates they core OTU was not identified to that taxonomic rank. The final column represents a mean of all studies.

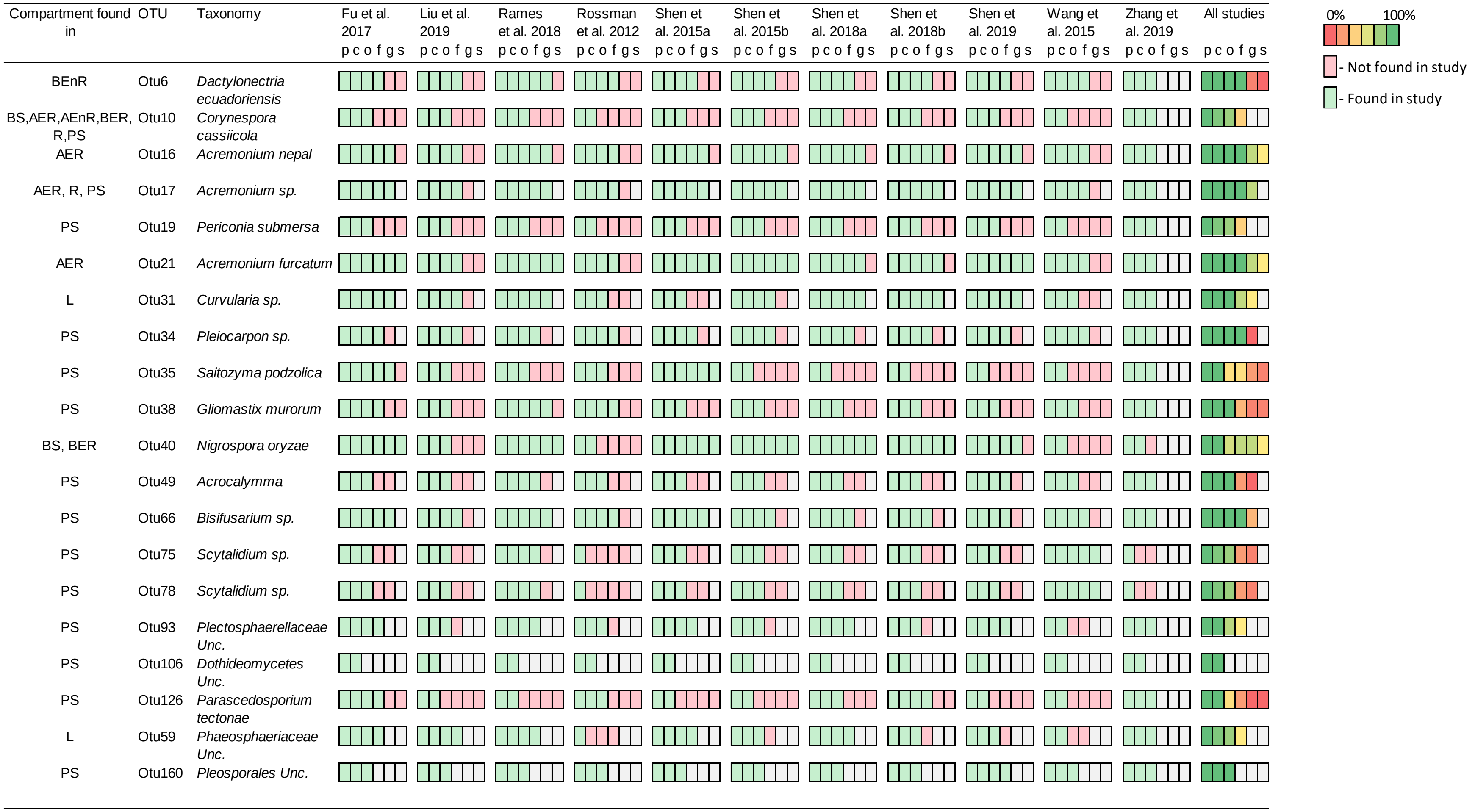

Fig S11 The presence of candidate-core OTUs in the taxonomy assigned to the top 10% of OTUs by maximum abundance in publicly available datasets that examine fungi associated with Musa spp. Green tiles represent presence of an OTU in a study with the same taxonomy as a core OTU, red indicates they were not present, white indicates they core OTU was not identified to that taxonomic rank. The final column represents a mean of all studies.
