# Supplementary material for: The core fungal microbiome of banana (Musa spp.)
Source: Front Microbiol. 2023 Mar 30;14:1127779. doi: 10.3389/fmicb.2023.1127779 (PMC10098452; doi:10.3389/fmicb.2023.1127779)
Supplement: Supplementary file 2 [file Data_Sheet_2.docx]

Supplementary experiment:

Testing of semi-nested PCR approach to prevent host contamination of ITS reads.

**The Core Fungal Microbiome of Banana Plants (*Musa* spp.)**

Henry W. G. Birt^1^, Anthony B. Pattison^2^, Adam Skarshewski^1^, Jeff Daniells^2^, Anil Raghavendra^1^, Paul G. Dennis^1*^

^1^*School of Earth and Environmental Sciences, The University of Queensland, Brisbane, QLD 4072, Australia;* ^2^*Department of Agriculture and Fisheries, Centre for Wet Tropics Agriculture, 24 Experimental Station Road, South Johnstone, QLD 4859, Australia.* *Correspondence: p.dennis@uq.edu.au

**Background**

To profile fungal communities, the ITS regions of rRNA operons are often amplified via polymerase chain reaction (PCR), sequenced and compared to a reference database. For host-associated fungal communities it is important to consider whether the PCR primers used to characterise fungi are likely to also amplify host DNA. To profile fungal communities, we typically use primers gITS7 (Ihrmark et al., 2012) and ITS4 (White et al., 1990), which amplify the ITS2 region. Experience, however, demonstrated that these primers co-amplify *Musa* spp. DNA. Hence, we designed a ‘semi-nested’ two-step PCR that avoids significant co-amplification of banana DNA. Here, we demonstrate using soil samples free of host-contamination, that the diversity of fungal communities recovered using this two-step PCR, is equivalent to that recovered using a single-step PCR with gITS7 and ITS4.

**Method**

Two sets of PCR conditions were tested to determine whether any bias was introduced through the semi-nested approach. The first used the semi-nested as described in the main text: ‘*An initial round of fifteen cycles of PCR was conducted using ITS-F_KYO1 (5’- CTH GGT CAT TTA GAG GAA STA A-‘3) (Toju et al., 2012) and ITS4 (5’- TCC TCC GCT TAT TGA TAT GC-‘3) (White et al., 1990). The resulting amplicons were then purified using magnetic beads (Rohland and Reich, 2011). Purified amplicons were used in a further twenty rounds of PCR using the primers gITS7 (5’- GTG AAT CAT CGA ATC TTT G-‘3) (Ihrmark et al., 2012) and ITS4 modified on the 5’ end to contain the Illumina overhang adapter for compatibility with the P5 and i7 Nextera XT indices, respectively. PCRs totalled 35 cycles. Thermocycling conditions were as follows: 98°C for 45 sec; then 35 cycles of 98°C for 5 sec, 56°C for 5 sec, 72°C for 6 sec; followed by 72°C for 1 min. Amplifications were performed using a Simpliampi® 96-well Thermocycler (Applied Biosystems). All PCRs were performed on 2 µl template in 5X Phire Green Reaction Buffer (Thermo Fisher), 100 µM of each dNTP (Invitrogen), 0.4 µl Phire Green Hot Start II DNA Polymerase (Thermo Fisher), 10 mM of each primer, made up to a total volume of 20 µl with molecular biology grade water*.’ This approach was used as the ITS-F_KYO1 primer was found to have far fewer matches to the rDNA of banana, when compared *in silico* through publicly available data downloaded from NCBI. The second set of PCR conditions were identical as above except for just a single round of 35 cycles using the gITS7 and ITS4 primers were used, representing a standard PCR.

Fifteen basal ectorhizosphere samples were chosen to be amplified and sequenced using both methods. These samples were chosen as little host contamination would be present compared to endophytic communities and therefore a standard PCR approach could be used to compare to the semi-nested approach.

The amplicons were sequenced and passed through the bioinformatic pipeline as described in the main text except for rarefaction of reads to 1500. Procrustes analysis was performed to determine the shift of samples resulting from the semi nested approach. This analysis was performed in R using the *vegan* package.

**Results and Discussion**

According to Procrustes analysis the ordination from the semi-nested PCR approach was highly correlated (*P* < 0.001) with the ordination of the standard PCR approach. While some drift of samples in ordination space was observed, this could easily be the result of stochastic change introduced from rarefaction, general amplification, and sequencing. From these results we determined that the semi-nested approach was appropriate for use in the main study.


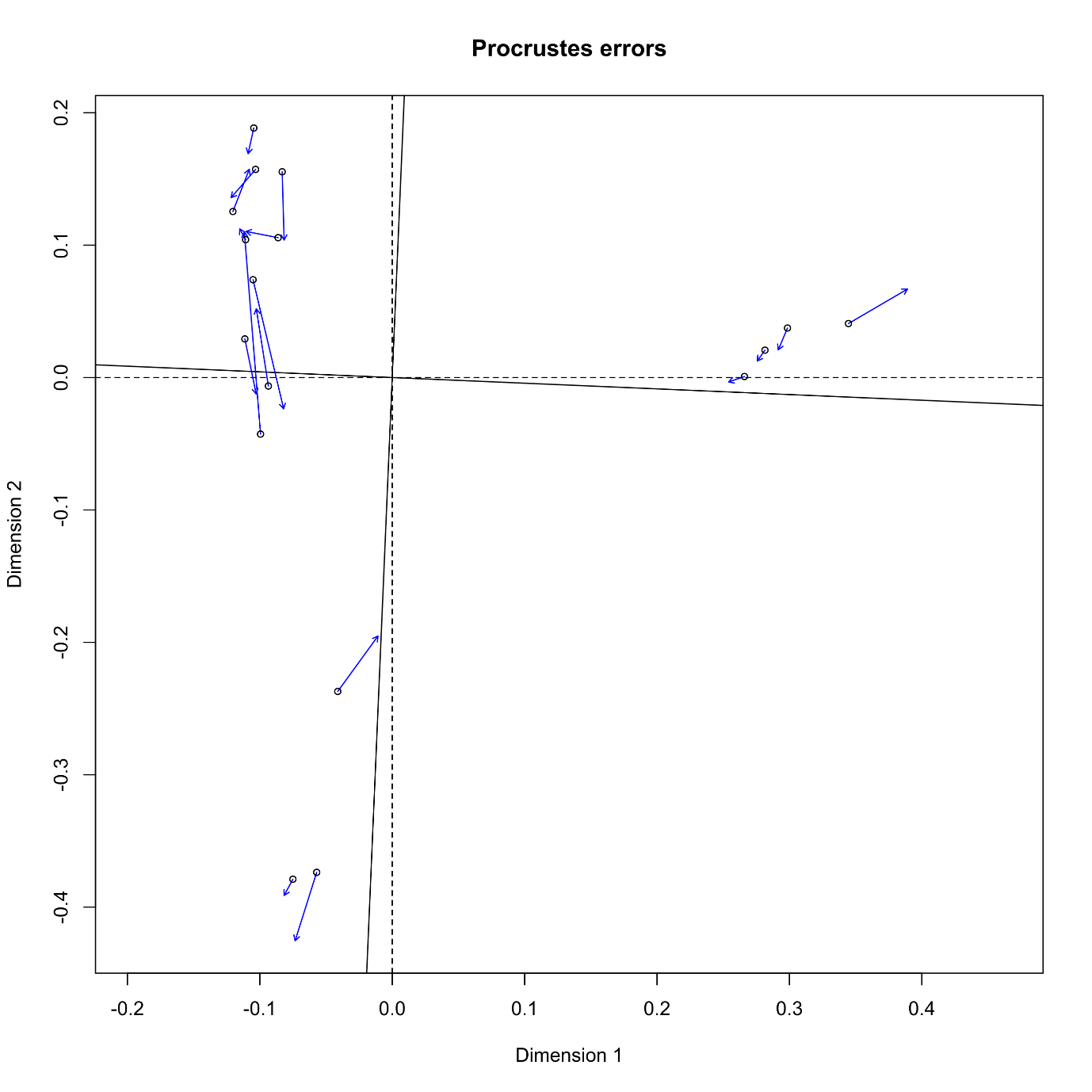


**Figure 1** The Procrustes rotation of two principal coordinate ordinations, the first representing a standard ITS PCR approach and the second a novel semi-nested ITS approach to reduce host contamination in the resulting sequencing reads.
